# Neuronal microstructural changes in the human brain are associated with neurocognitive aging

**DOI:** 10.1111/acel.14166

**Published:** 2024-04-24

**Authors:** Kavita Singh, Stephanie Barsoum, Kurt G. Schilling, Yang An, Luigi Ferrucci, Dan Benjamini

**Affiliations:** ^1^ Multiscale Imaging and Integrative Biophysics Unit National Institute on Aging, NIH Baltimore Maryland USA; ^2^ Department of Radiology and Radiological Sciences Vanderbilt University Medical Center Nashville Tennessee USA; ^3^ Brain Aging and Behavior Section National Institute on Aging, NIH Baltimore Maryland USA; ^4^ Translational Gerontology Branch National Institute on Aging, NIH Baltimore Maryland USA

**Keywords:** aging, diffusion, gray matter, microstructure, MRI, neurocognitive aging

## Abstract

Gray matter (GM) alterations play a role in aging‐related disorders like Alzheimer's disease and related dementias, yet MRI studies mainly focus on macroscopic changes. Although reliable indicators of atrophy, morphological metrics like cortical thickness lack the sensitivity to detect early changes preceding visible atrophy. Our study aimed at exploring the potential of diffusion MRI in unveiling sensitive markers of cortical and subcortical age‐related microstructural changes and assessing their associations with cognitive and behavioral deficits. We leveraged the Human Connectome Project‐Aging cohort that included 707 participants (394 female; median age = 58, range = 36–90 years) and applied the powerful mean apparent diffusion propagator model to measure microstructural parameters, along with comprehensive behavioral and cognitive test scores. Both macro‐ and microstructural GM characteristics were strongly associated with age, with widespread significant microstructural correlations reflective of cellular morphological changes, reduced cellular density, increased extracellular volume, and increased membrane permeability. Importantly, when correlating MRI and cognitive test scores, our findings revealed no link between macrostructural volumetric changes and neurobehavioral performance. However, we found that cellular and extracellular alterations in cortical and subcortical GM regions were associated with neurobehavioral performance. Based on these findings, it is hypothesized that increased microstructural heterogeneity and decreased neurite orientation dispersion precede macrostructural changes, and that they play an important role in subsequent cognitive decline. These alterations are suggested to be early markers of neurocognitive performance that may distinctly aid in identifying the mechanisms underlying phenotypic aging and subsequent age‐related functional decline.

## Graphical abstract

Leveraging the Human Connectome Project‐Aging cohort with 707 participants, our study revealed strong age‐related associations with both macro‐ and microstructural gray matter changes. Unlike macrostructural volumetric changes, microstructural alterations in cellular morphology and density were significantly correlated with cognitive function. These microstructural changes may serve as early markers of neurocognitive decline in aging.

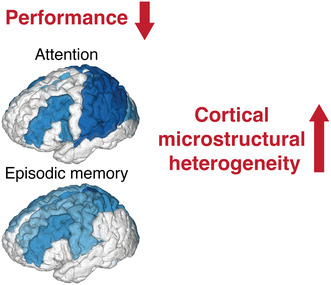

**Abbreviations**
AD: axial diffusivity
ant: anterior
dMRI: diffusion MRI
DTI: diffusion tensor imaging
DWI: diffusion‐weighted images
FA: fractional anisotropy
FDR: false discovery rate
G: gyrus
GM: Gray matter
HCP‐A: Human Connectome Project‐Aging
ICV: intracranial volume
inf: inferior
MAP: mean apparent propagator
MoCA: Montreal Cognitive Assessment
NG: non‐Gaussianity
PA: propagator anisotropy
post: posterior
PSM: picture sequence memory
RAVLT: Rey auditory verbal learning test
RD: radial diffusivity
ROI: region of interest
RTAP: return to the axis probability
RTOP: return to the origin probability
RTPP: return to the plane probability
SLANT: spatially localized atlas network tiles
sup: superior
TR: diffusion trace
WM: white matter
YOE: years of education

## 1 INTRODUCTION

Magnetic resonance imaging (MRI) macrostructural volumetric studies focused on the gray matter (GM) have demonstrated consistent correlations between older age and longitudinal decline of cortical volume and thickness (Sowell et al., 2003). Though, spatial localization and degree of atrophy are not homogeneous across the aging brain, and for example, frontal and temporal lobes exhibit the highest degree of atrophy, parietal lobe exhibits moderate changes, whereas the occipital lobe appears to remain relatively intact (Peters, 2006). At the macroscopic level, the occurrence and extent of age‐related volumetric changes of white matter (WM) show variability and inconsistency (Gunning‐Dixon et al., 2009). However, diffusion MRI (dMRI), which probes meso‐ and micro‐structural information (Callaghan, 2011), enables the investigation of age‐related changes in individual fiber pathways of the brain that are not detected by traditional MRI.

Diffusion tensor imaging (DTI) (Basser et al., 1994), which describes the distribution of diffusion displacements using a simplistic Gaussian model, is the most widely used dMRI experimental and theoretical framework to study the microstructural integrity of brain tissue. Studies using DTI have unveiled complex and nonlinear age‐related patterns in brain tissue during maturation and degeneration, typically reflecting increases in anisotropy and decreases in diffusivity during childhood, adolescence, and early adulthood, and subsequent anisotropy decreases, and diffusivity increases in adulthood and senescence (Schilling et al., 2022). Nonetheless, contrary to basic assumptions in DTI, diffusion within heterogeneous biological tissues is non‐Gaussian, primarily due to the complex microstructure involving the presence of cell membranes, organelles, and distinct liquid compartments. Consequently, while DTI exhibits considerable structural sensitivity, it is hindered by these limitations and by its lack of specificity, which in turn impede its interpretation. To enhance specificity, various multicomponent biophysical models have been developed (Zhang et al., 2012), some of which have been used to study aging (Nazeri et al., 2015). While proving effective in providing interpretable metrics of cortical alterations (Gozdas et al., 2021; Vogt et al., 2020), these models were created for axonal microstructure, and as a result, their limitations in studying GM due to deviations from assumed conditions are well‐documented (Kundu et al., 2023; Palombo et al., 2020). Consequently, to date, most dMRI microstructural studies on brain aging have predominantly concentrated on WM.

The importance of developing a sensitive MRI framework to assess age related microstructural changes in GM has been highlighted by recent anti‐amyloid‐β clinical trials that showed increased GM atrophy was observed despite evidence of target engagement and a slowdown in cognitive decline (Mintun et al., 2021). Such outcomes may be attributed to underlying micro‐ and meso‐structural changes, reflecting alterations in axonal connectivity, cellular morphology, and the neuroinflammatory response in GM. Although few studies suggested that dMRI is sensitive to cortical microstructural changes in unimpaired and impaired aging, they were limited to large GM regions and were performed on relatively small cohorts (Gozdas et al., 2021; Nazeri et al., 2015). A recent pilot study showed that the mean apparent propagator model (MAP‐MRI) model (Özarslan et al., 2013), a dMRI experimental and theoretical framework which does not rely on any biophysical assumptions, can leverage multi‐shell dMRI data and provide high sensitivity to age‐related microstructural changes in GM (Bouhrara et al., 2023). Echoing these results, cortical microstructural alteration derived from MAP‐MRI were shown to be highly sensitive to multiple aspects of the Alzheimer's disease pathological cascade (Spotorno et al., 2023). Indeed, MAP‐MRI, which can be thought of as a generalization of DTI, may facilitate the study of healthy and diseased white and gray matter as it directly measures the diffusion propagator in each voxel, enabling comprehensive insights into tissue diffusion processes and microstructural parameter derivation, unconstrained by assumptions about tissue compartments that are unlikely to hold in normal aging and disease.

In this work, we use MAP‐MRI to probe cerebral GM microstructure and architecture in the large cohort of the Human Connectome Project‐Aging (HCP‐A). This represents the most in‐depth application of MAP‐MRI in normal aging to date, incorporating state‐of‐the‐art processing procedures to allow microstructural investigation of cortical and subcortical GM regions. In this study, we aim to: (1) Characterize GM macro‐ and microstructural differences with age using volumetric and MAP‐MRI, respectively; (2) investigate associations of GM macro‐ and microstructure with cognitive measures and determine whether one or both can be used as predictors of neurobehavioral performance; and (3) compare MAP‐MRI age‐ and cognitive associations with those obtained from DTI.

## 2 RESULTS

**FIGURE 1:**
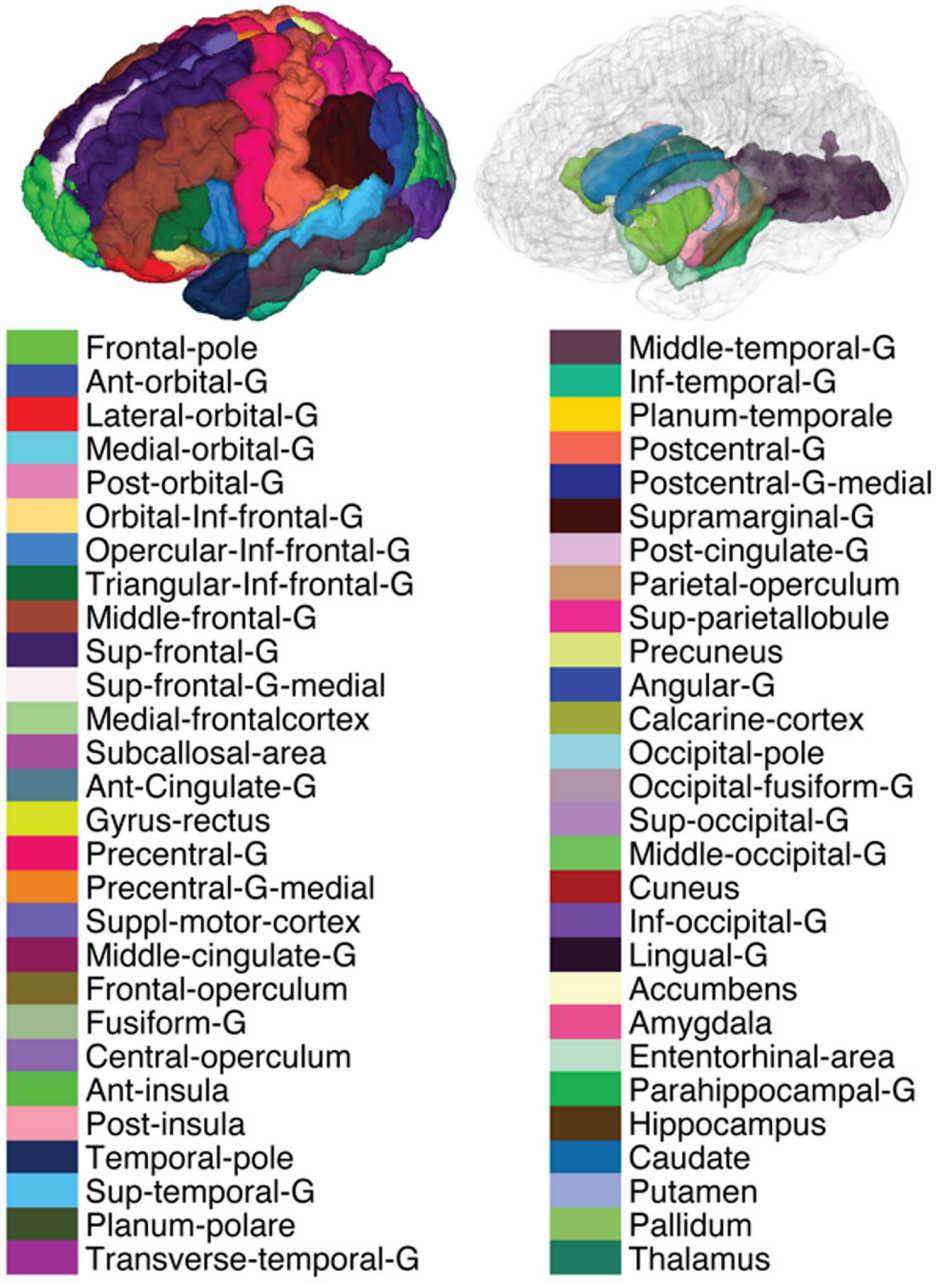
Cortical and subcortical view of the 56 brain regions of interest (ROI) used in the study. Note that the ROI‐based analysis was performed in the native space of each subject to minimize errors resulting from interpolation. Ant, anterior; G, gyrus; Inf, inferior; Post, posterior; Sup, superior.

Imaging data from 707 subjects aged 36–90 years were included in this study, along with their available behavioral and cognitive test scores (Bookheimer et al., 2019). The list of tests and respective scores stratified by participants age is provided in Table 1. A total of 56 cortical and subcortical GM regions of interest (ROIs), covering most of the brain, were included in the study (Figure 1). For each MRI metric and ROI, only statistically significant associations that survived multiple comparison correction were shown (*p*_FDR_ <0.05), where *β*_age_ expresses the rate of change from young to adult (at sample's mean age of 59.6 years), *β*_age_^2^ reflects the direction and steepness of the quadratic association, and *β*_MR_ reflects the rate of change of the various MR metrics with respect to the different neurobehavioral test scores. Further details about the statistical models can be found in the Section 4.

**TABLE 1:** Participant demographics (*N* = 707) and list of neurobehavioral test scores expressed as mean (standard deviation).

	All	Age ≤60	Age >60	*p* Value*	Missing samples	Neurobehavioral domain
*N*	707	383	324			–
Age [years]	59.7 (14.9)	47.9 (7.0)	73.6 (8.3)	<0.001	0	–
Sex, F/M	394/313	221/162	173/151	0.251	0	–
YOE [years]	17.5 (2.2)	17.4 (2.2)	17.6 (2.1)	0.116	1	–
Flanker	8.00 (0.82)	8.26 (0.73)	7.69 (0.82)	<0.001	86	Executive function, attention
PSM	505 (103)	551 (92.7)	448 (86.0)	<0.001	85	Episodic memory
Trail Making A	29.9 (11.8)	26.3 (10.1)	34.1 (12.3)	<0.001	5	Cognitive processing speed
Trail Making B	74.1 (41.9)	64.5 (34.6)	85.4 (46.9)	<0.001	3	Executive functioning
RAVLT 1	−0.32 (0.47)	−0.26 (0.41)	−0.40 (0.52)	<0.001	10	Episodic memory (retroactive)
RAVLT 2	−0.15 (0.60)	−0.08 (0.50)	−0.24 (0.69)	<0.001	7	Episodic memory (proactive)

*p values were derived from two sample *t*‐test for continuous variables and chi‐square test for categorical variables.

Abbreviations: PSM, Picture Sequence Memory; RAVLT, Rey Auditory Verbal Learning Test; YOE, years of education.

The MAP‐MRI framework does not rely on any assumptions and is suitable to use in complex microstructures, offering consequential advantages over biophysical model‐based dMRI methods. While MAP‐MRI provides five distinct microstructural metrics, namely, return to the axis/origin/plane probabilities—RTAP, RTOP, RTPP (jointly referred to as the zero‐displacement probabilities), propagator anisotropy (PA), and non‐Gaussianity (NG), they do not yield straightforward structural metrics and requires interpretation. The zero‐displacement probability variables were shown to be proxies of cellular density and extracellular volume (Avram et al., 2022), the PA quantifies variations in water diffusion along different orientations, and the NG reflects microstructural heterogeneity (i.e., the presence of microenvironments with different diffusivities) (Avram et al., 2016). Representative examples of these MAP‐MRI maps are shown in Figure S1. For completeness, we analyzed DTI metrics as well.

All MAP‐MRI, DTI, and volumetric indices as a function of age, Flanker, PSM, Trail Making A, Trail Making B, RAVLT1, and RAVLT2 test scores from four representative ROIs are shown in Figures S2–S5. Visual inspection indicates quadratic age relation with age of all diffusion indices, although the rates of maturation or degeneration appear to be different. Below, we assessed the effect of age on MRI parameters using an age quadratic model, which captures both linear and nonlinear age associations (see Section 4).

### 2.1 Linear associations between age and regional GM macro‐ and microstructure

**FIGURE 2:**
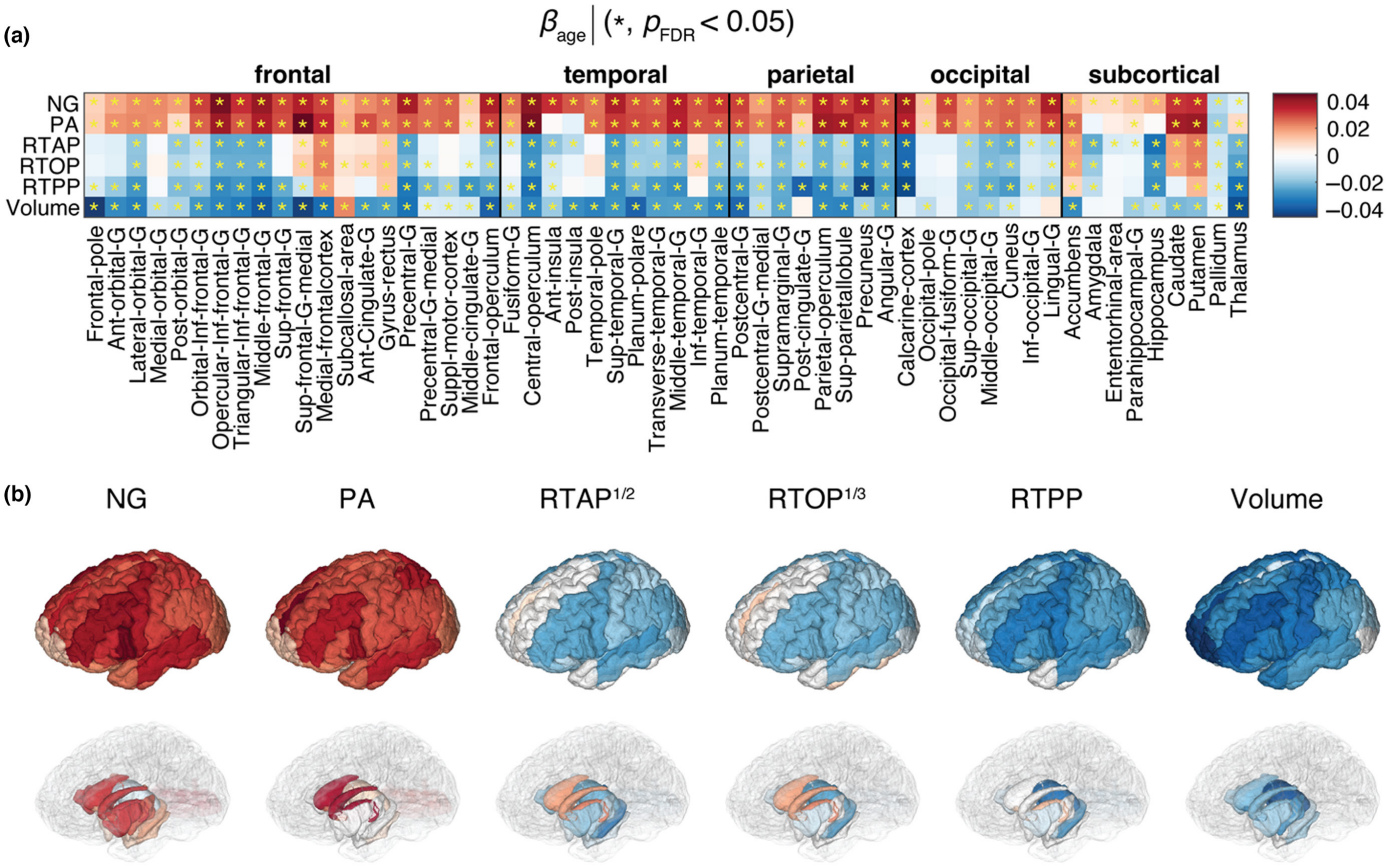
Linear associations of MAP‐MRI and volumetric metrics and age. (a) The *β*_age_ coefficients are shown as a matrix for MAP‐MRI and volumetric z‐normalized features across all 56 ROIs. Blocks marked with an asterisk (*) represent associations meeting the *p*_FDR_ <0.05 threshold. (b) 3D visualization of significant results in cortical (top) and subcortical (bottom) view.

We first examined the linear associations with age (at centered age of 59.6 years) of several MRI parameters expressed by the *z*‐scored (standardized) *β*_age_ in our regression model. Macro‐ and microstructure in GM were captured by ROI volume and MAP‐MRI parameters, respectively (Figure 2). After correction for multiple comparisons, most of the cortical and subcortical ROIs for all the parameters were significantly associated with age. Microstructurally, most of the regions showed positive associations of NG and PA and negative associations of RTAP, RTOP, and RTPP and age. However, some regions, for example, the medial frontal cortex, gyrus rectus, accumbens, caudate, and putamen, exhibited opposing trends in the RTAP, RTOP, and RTPP metrics. Macrostructurally, negative correlation of ROI volume and age was generally observed.

The linear associations of DTI parameters with age in GM are shown in Figure S6. Relative to the other microstructural metrics, the fractional anisotropy (FA) exhibited lower age‐related sensitivity. Of note are the opposing trends of FA and PA with respect to age, indicating that although both parameters measure diffusion anisotropy, they provide different information. The age associations of axial diffusivity (AD), radial diffusivity (RD), and diffusion trace (TR) followed their MAP‐MRI counterparts (RTAP, RTOP, and RTPP), only with an inverse relationship, as expected (Bouhrara et al., 2023).

### 2.2 Quadratic associations between age and regional GM macro‐ and microstructure

**FIGURE 3:**
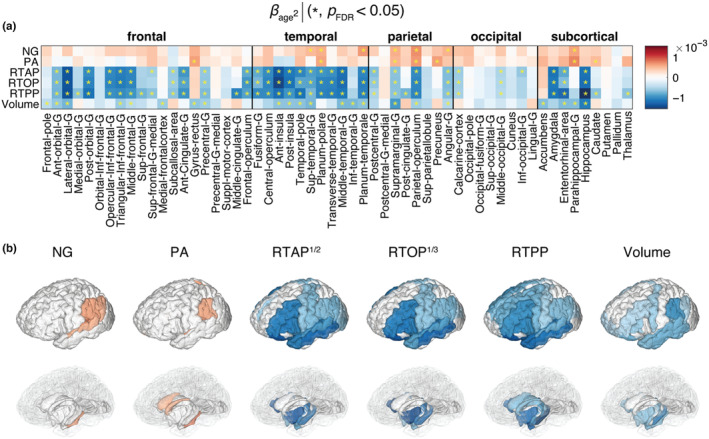
Quadratic associations of MAP‐MRI and volumetric metrics and age. (a) The *β*_age_^2^ coefficients are shown as a matrix for MAP‐MRI and volumetric z‐normalized features across all 56 ROIs. Blocks marked with an asterisk (*) represent associations meeting the *p*_FDR_ <0.05 threshold. (b) 3D visualization of significant results in cortical (top) and subcortical (bottom) view.

Nonlinear age relationships in GM are characterized by the direction and steepness of the quadratic curvature (z‐scored *β*_age_^2^), as depicted in Figure 3. However, not all GM regions survived multiple comparisons correction, and specifically, NG and PA did not display significant quadratic age relationships in most ROIs. The prominent negative associations observed with RTAP, RTOP, and RTPP, which are sensitive to cellular density and extracellular volume, were primarily concentrated in frontal, parietal, and temporal regions, including the lateral orbital gyrus, posterior‐orbital gyrus, anterior insula, medial temporal gyrus, planum temporale, supramarginal gyrus, and parietal operculum, as well as in the medial temporal gyrus, entorhinal area, parahippocampus, hippocampus, and amygdala. While occipital regions yielded fewer significant results, the angular gyrus and calcarine cortex stood out. Importantly, subcortical regions and memory‐related areas such as the hippocampus, known for their vulnerability in age‐related cognitive decline disorders, demonstrated significant inverted U‐shape associations with age. Macrostructurally, inverted U‐shape quadratic correlation of ROI volume and age was observed in the frontal, parietal, temporal, subcortex, and limbic system regions.

The nonlinear associations of DTI parameters with age in GM are shown in Figure S7. Similar to NG and PA, the FA in most cortical and subcortical regions did not exhibit a significant quadratic association with age. Strong U‐shape quadratic age associations were observed with the AD, RD, and TR, as previously reported (Bouhrara et al., 2023).

### 2.3 Macro‐ and microstructural age trajectories

**FIGURE 4:**
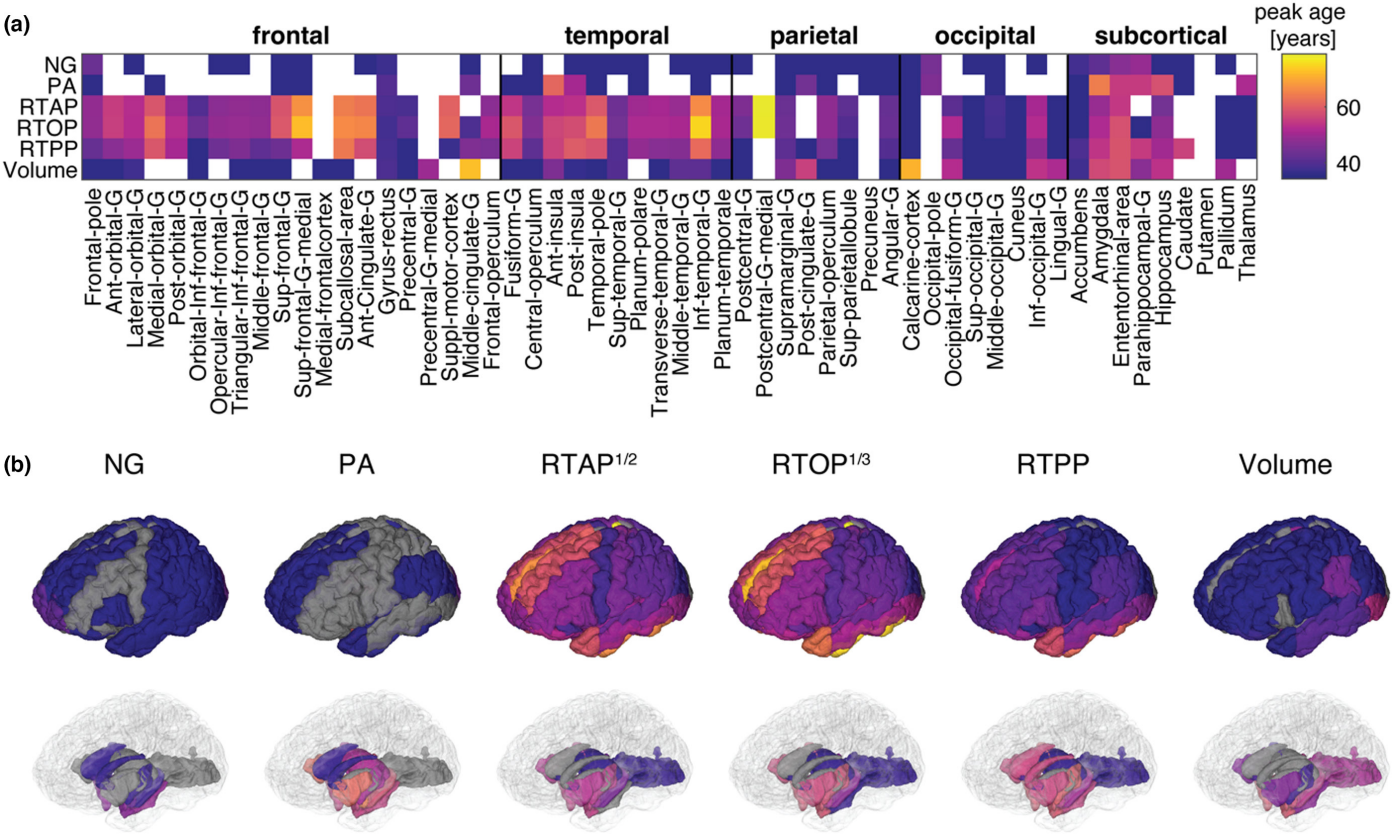
Peak ages for MAP‐MRI and volumetric within each ROI. (a) Matrix representation of peak age across all 56 ROIs. (b) 3D visualization of significant peak ages in cortical (top) and subcortical (bottom) view.

The peak ages for each MR metric within each ROI are illustrated in Figure 4. To avoid extrapolation beyond the extent of our data, we left censored the peak age at 36, omitted peak ages over 80 years and negative values. As anticipated, the lack of significant quadratic age associations for NG and PA in most ROIs is consistent with the absence of plausible peak age values in some of those regions. The median standard error across all ROIs was 11.0, 9.4, 3.2, 3.4, 3.0, and 6.0 years for NG, PA, RTAP, RTOP, RTPP, and volume, respectively. When considering peak age as associated with onset of age‐related changes, we observed diverse trends.

Microstructurally, MAP‐MRI metrics revealed varying peak ages across different brain regions, indicating the spatial distribution of age‐related changes, as well as their onset. The NG and PA generally showed the earliest peaks, which could point to early onset of increased microstructural heterogeneity and reduced neurite orientation dispersion, respectively. On the contrary, the zero‐displacement probabilities exhibited considerably later peak ages in the late 40's, indicating that reduced cellular density and increased extracellular volume may exacerbate at a later stage in life. Macrostructurally, peak age with respect to volumetric changes in most cortical regions demonstrated later onset compared with NG and PA, and earlier onset compared with the zero‐displacement probabilities. Memory‐related subcortical regions like the parahippocampus and hippocampus peaked at a relatively older age (about 50 years).

Examining the peak ages from DTI‐derived AD, RD, and TR parameters reveals trends similar to those observed from MAP‐MRI derived the zero‐displacement probabilities (Figure S8). The FA demonstrated later onset compared with the other DTI and MAP parameters.

Further evidence supporting NG and PA as early markers of age‐related microstructural changes is given by examining their linear associations with age, specifically within a younger subset of the cohort (age < 50 years, *N* = 227). This analysis showed significant associations, primarily with NG and PA, in numerous frontal, temporal, parietal, occipital, and subcortical brain regions (Figure S9). Similar analysis applied towards DTI metrics showed no significant associations in any regions, except for the caudate and putamen (Figure S10).

### 2.4 Micro‐ and not macrostructure is associated with neurobehavioral performance

**FIGURE 5:**
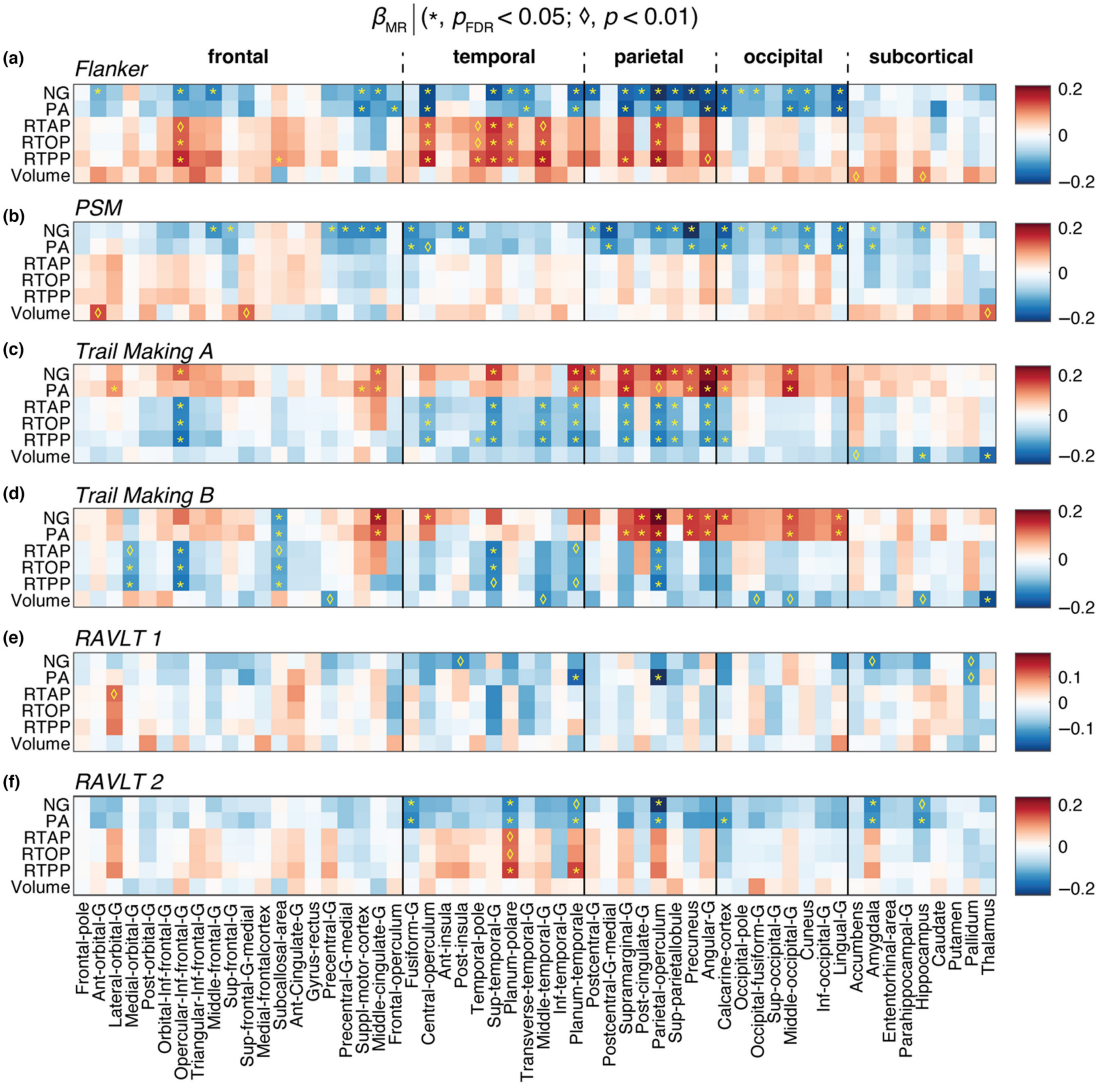
Significant results of second statistical model examining the relationship between the macro‐ and microstructural MRI metrics and six cognitive test scores by modeling them as outcomes, adjusted for age, sex, and years of education. Significant associations are expressed using the regression parameters *β*_MR_. Blocks marked with an asterisk (*) represent associations meeting the *p*_FDR_ <0.05 threshold. Blocks marked with a diamond (♢) represent associations meeting the *p* < 0.01 threshold without FDR correction. For all tests, the lower the score the worse the performance, except for the Trail Making task, in which the opposite is true. PSM, Picture Sequence Memory test; RAVLT, Rey auditory verbal learning test.

**FIGURE 6:**
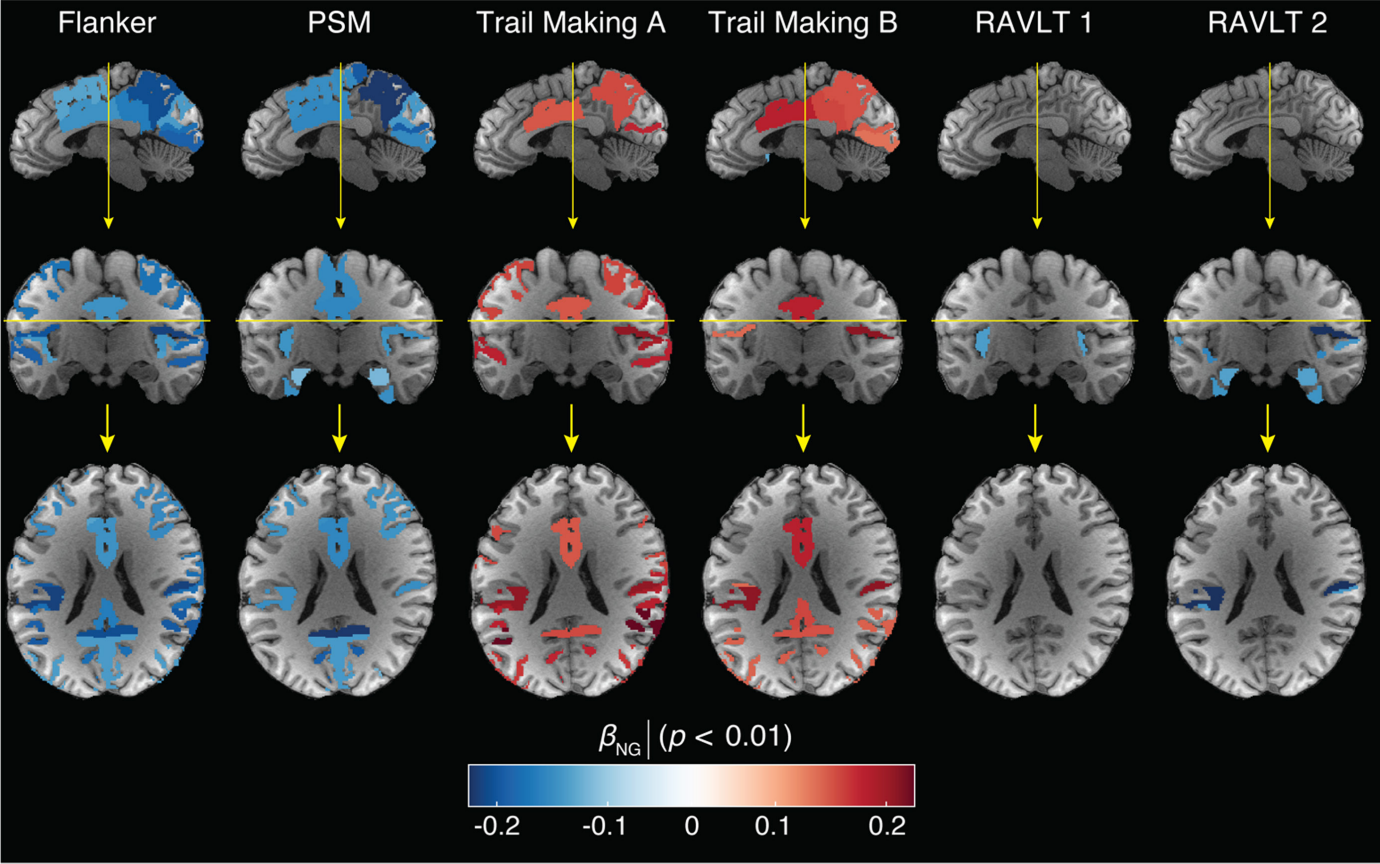
Axial, coronal, and sagittal views of significant results of relationship between the non‐Gaussianity (NG) and six cognitive test scores. Age‐related significant associations are expressed using the regression parameters *β*_NG_ with *p* < 0.01. For all tests, the lower the score the worse the performance, except for the Trail Making task, in which the opposite is true. PSM, Picture Sequence Memory test; RAVLT, Rey auditory verbal learning test. Yellow lines indicate sectional planes.

We proceeded to examining the relationship between the macro‐ and microstructural MRI metrics and cognitive test scores by modeling them as outcomes, adjusted for age, sex, and education. Figure 5 shows the linear association of different MR metrics as expressed by z‐scored *β*_MR_ with respect to the Flanker, PSM, Trail Making, and RAVLT tests scores. Interestingly, almost exclusively the NG and PA metrics resulted in significant correlations with the various cognitive and behavioral test scores that survived multiple comparisons correction. These metrics, which relate to microstructural heterogeneity and to neurite orientational dispersion, respectively, were also found to exhibit the earliest onset of age‐related changes. The NG appeared to be the most robust MAP‐MRI predictor towards cognitive performance, and therefore its correlation coefficient with respect to the tests, *β*_NG_, is shown in sagittal, coronal, and axial views in Figure 6 to provide visualization of its spatial distributions.

Linear associations of DTI metrics with respect to cognitive performance test scores are shown in Figures S11 and S12. Compared with MAP‐MRI, DTI‐based diffusivities measures exhibited significant associations in largely the same regions as the MAP‐derived zero‐displacement probabilities, thus indicating overlap of these parameters. However, the FA was not found significant in any of the ROIs or neurocognitive performance (except for four regions with respect to the Flanker score).

The Flanker inhibitory control task, a measure of executive function and attention where lower scores indicate poorer performance, is known to exhibit functional associations with the cingulo‐opercular and fronto‐parietal control networks (Dosenbach et al., 2007). Our findings reveal extensive and significant negative correlations of both NG and PA metrics in regions associated with these control networks, as visualized in Figures 5a and 6a. Positive associations with the zero displacement probabilities were observed in temporal and parietal regions.

The PSM task score assesses episodic memory, in which the lower the score the worse the performance, exhibited significant negative correlations with NG and PA in brain regions responsible for encoding new learning into episodic memory, which encompassed primary sensory areas (visual, auditory, and somatosensory) and memory‐related regions (prefrontal cortex, amygdala, and hippocampus), as shown in Figures 5b and 6. Notably, significant negative correlations were observed in several brain regions, including the frontal, temporal, parietal, and occipital cortical regions, as well as the amygdala and hippocampus.

The Trail Making tests evaluate task completion time, examining psychomotor speed, visuospatial search, and target‐directed motor tracking that are linked to prefrontal and parietal cortices (Varjacic et al., 2018). As such, the higher the score the worse the performance. In our study, the Trail Making A test significantly correlated with NG and PA in network regions like the lateral orbital gyrus, planum temporale, angular gyrus (related to attention, self‐processing), and visual network regions including the middle occipital gyrus, depicted in Figures 5c and 6. Trail Making B test scores, which measure executive functioning, were significantly correlated with NG and PA in the parietal cortical regions.

The RAVLT test, with variants 1 and 2, assesses episodic memory function. Lower scores indicate poorer performance. RAVLT 1 relates to retroactive interference and was negatively correlated with PA in planum temporale and parietal operculum, areas linked to auditory processing and multimodal integration, respectively. RAVLT 2 relates to proactive interference and showed widespread correlations with PA, NG, and RTPP in proactive interference mediating regions involved in memory, including the posterior parietal cortex, medial‐temporal lobe, occipital regions, amygdala, and hippocampus (Straube, 2012).

Importantly, macrostructural volumetric information was not predictive of neurobehavioral performance in any of the tests, with the exception of the thalamus in the Trail Making tests.

Effect sizes are provided as partial correlation coefficients for all the above analyses and are summarized in Figures S13 and S14.

## 3 DISCUSSION

Gray matter alterations have been shown to be associated with aging and various aging‐related disorders such as Alzheimer's, Parkinson's, and dementias. Examinations of aging‐related brain changes using MRI have predominantly focused on the macroscopic level in GM or employed DTI to study WM microstructure. With the advent of advanced models like MAP‐MRI, microstructural changes in GM can now be studied without violating the model's assumptions. By conducting an extensive analysis of a large, cross‐sectional dataset from the Human Connectome Project encompassing 707 subjects aged between 36 and 90 years, we examined MAP‐ and DTI‐derived micro‐ and volumetric‐derived macrostructural features, in addition to cognitive and behavioral metrics, across 56 cortical and subcortical brain regions, and their relationships with age. Notably, while volumetric MRI did not display associations with age‐related cognitive and behavioral performance, microstructural features exhibited strong correlations with memory and executive functions in the corresponding GM regions. Expanding on our previous study, where we demonstrated the superiority of MAP‐MRI over DTI in assessing GM (Bouhrara et al., 2023), we established here that this superiority holds true in the HCP cohort as well. We then presented an in‐depth and comprehensive characterization of age‐related microstructural alterations in GM using MAP‐MRI (and DTI for comparison) across the adult lifespan and elucidated their associations with cognitive and behavioral performance.

Considering MAP‐MRI as a generalization of DTI enables a direct understanding of both the additional information it offers and its limitations. While biophysical model‐based dMRI methods provide more readily interpretable metrics than MAP‐MRI, the latter operates without assumptions and is suitable to use in complex microenvironments, while circumventing the low b‐value range limitation of DTI. To bridge the gap between MAP‐MRI metrics and biophysical interpretation, we assume here that the zero‐displacement probabilities (RTAP, RTOP, and RTPP) are inversely related to the nominal size of microstructures and are affected by cellular density and extracellular volume; the PA captures the three‐dimensional directionality of diffusion, and in GM it encompasses information about microstructural shape and neurite orientation dispersion; and the NG quantifies the deviation from DTI's simple tensor model and reflects the heterogeneity of microstructures.

### 3.1 Gray matter macro‐ and microstructural changes with age: Linear and nonlinear associations

When examining the age‐related linear and quadratic trends of MAP‐MRI features, we identified positive relationships with PA and NG, and negative correlations with zero‐displacement probabilities in most GM regions (see Figures 2 and 3). Our findings established that the associations between NG and PA with age in GM were primarily linear, while the zero‐displacement probabilities displayed substantial quadratic associations in most GM regions, except for the occipital regions. Additionally, we observed macrostructural changes involving volumetric reductions in most regions. Much like the results from MAP‐MRI, the volumetric reductions in occipital regions did not exhibit significant quadratic associations with age.

The negative quadratic associations with age trends we observed with respect to the zero displacement probabilities are in agreement with a previous MAP‐MRI pilot study (Bouhrara et al., 2023), and also with the widely observed behavior of diffusivity metrics in GM across the adult lifespan (Schilling et al., 2022). These results can be hypothesized to indicate reduced cellular density and increased extracellular volume in GM with age. In addition, local inflammation, which is a common feature of aging brain, leads to activated state of microglia and astrocytes in the aged brain. The resulting glial morphological change, in particular astrocytes, which includes hypertrophy of the cell body and stem processes (Sofroniew, 2020), is therefore hypothesized to contribute as well to the decrease of MAP‐MRI measured zero displacement probabilities with age.

The PA provides a higher‐order assessment of DTI's FA and quantifies the directional dependence of the diffusion process. From our analysis, we found that the PA is positively linearly associated with age in almost all GM regions. While age‐related patterns of increased anisotropy during childhood, adolescence, and early adulthood, and subsequent anisotropy decrease in adulthood and senescence (Schilling et al., 2022), have been observed in WM, increased FA with age in deep brain GM structures has been previously reported (Pfefferbaum et al., 2010). Further, older age was associated with reduced neurite orientation dispersion in widespread cortical regions (Bouhrara et al., 2023; Gozdas et al., 2021). Thus, microstructurally, our findings with respect to PA are hypothesized to reflect morphological changes in neurons, which are expected to decrease in number, shorten, and become less branched with fewer spines (Dickstein et al., 2013).

The zero‐displacement probabilities and the PA extend the DTI model and its derived diffusivity parameters, providing metrics that could be more sensitive to subtle pathological events and more suitable to complex microstructure (Avram et al., 2016). On the contrary, the NG provides completely distinct information that quantifies the dissimilarity between the full propagator and its Gaussian component, and in essence reflects the deviation from DTI's tensor model. With that, while the NG is the least interpretable MAP‐MRI parameter, it carries unique information and also seems to be the most microstructurally sensitive with respect to the aging brain (Bouhrara et al., 2023). Similar to the PA in our study, we found significant positive linear associations of the NG with age in almost all GM regions. The non‐Gaussianity of the diffusion processes can be driven primarily by restriction (Benjamini & Basser, 2019), membrane permeability (Williamson et al., 2019), and cellular degradation (Benjamini et al., 2020), which together form microstructural heterogeneity. The robust increases of NG in normative aging that we observed supports the microstructural scenario of dendritic changes, reactive astrocytes, especially hypertrophy of stem processes, and increased membrane permeability due to myelin alterations through processes like demyelination and remyelination (Lee & Kim, 2022).

### 3.2 Trajectories of macro‐ and microstructural changes with age are heterogeneous

Although impossible to determine without a longitudinal study, information about the period at which age‐related micro‐ and macrostructural changes become apparent can be gleaned from estimating the peak age with respect to each MR parameter (Figure 4). While it cannot depict dynamic progression, the cross‐sectional peak age can provide useful information regarding the onset of age‐related changes and help elucidate the trajectories of different micro‐ and macrostructural changes. Our results showed diverse trends, in which some changes appear to precede others. Specifically, we found that the NG and PA reach peak age earliest, followed by macroscopic volume, and finally zero‐displacement probabilities. These results imply that increased microstructural heterogeneity and decreased neurite orientation dispersion (reflected from NG and PA, respectively) may precede macrostructural changes (reflected from regional volumes) and changes in cellular density and extracellular volume (reflected from RTAP, RTOP, and RTPP). Additional support for NG and PA as early indicators of age‐related microstructural changes is provided by analyzing their linear correlations with age specifically within a younger subset of the cohort, which revealed significant associations (Figure S9). Within the same cohort subset, DTI metrics have largely yielded insignificant age associations (Figure S10), pointing to potential increased sensitivity of MAP‐MRI to early age‐related microstructural changes.

Interestingly, regions associated with the limbic system, paralimbic areas, and limbic‐related regions like the anterior cingulate, amygdala, entorhinal area, parahippocampus, hippocampus, temporal pole, and cortex‐insula, exhibited the latest peak age based on both MAP‐MRI and volumetric analyses. These findings align with the concept of the limbic network displaying relative age resilience, given its role in emotions and memories. For instance, the limbic network is recognized for demonstrating minimal volumetric reductions in cognitively healthy aging populations (Fujita et al., 2023), and relatively stable DTI parameters in limbic‐related white matter tracts, such as the parahippocampal cingulum (Bennett et al., 2015). These studies consistently report the preservation, relative stability, and sparing of the medial temporal lobe in healthy aging, encompassing the entorhinal and parahippocampal cortices across all age groups. Our findings here align with these studies, demonstrating preserved micro‐ and macrostructural characteristics in the limbic network. It is hypothesized that cognitive reserve mechanisms might safeguard against changes in the micro‐ and macrostructure of the brain within the limbic network, a topic that warrants exploration in future research.

### 3.3 Gray matter microstructure linked to neurobehavioral performance

Significant associations between performance in various behavioral and cognitive tasks and microstructural changes, primarily reflected from NG and PA, were observed across several brain regions (Figures 5 and 6). Importantly, while we showed that volumetric and some DTI parameters are highly correlated with age in GM, these changes proved to be poor predictors of neurobehavioral function. These findings, combined with the earlier peak age of the NG and PA compared with volumetric changes, can be hypothesized to reflect that certain microstructural changes precede macrostructural ones, and that the former can predict the development of the latter. These results underscore the importance of quantifying microstructural and architectural features in GM in the context of aging.

Significant negative correlations of NG and PA were identified in regions crucial for executive function (fronto‐parietal control network) and attention (cingulo‐opercular network). The negative correlations of the Flanker score with PA reflects reduced neurite orientation dispersion in regions supporting cognitive control and visual processing speed, in agreement with reported reduction in structural and functional connectivity in aging (Ruiz‐Rizzo et al., 2019). Our study found broad associations between PSM task scores and NG and PA, highlighting the known sensitivity of episodic memory to cerebral aging (Nyberg et al., 2012). Negative associations between NG and PA values, reflecting reduced neurite orientation dispersion, were observed not only in memory‐related regions but also in primary sensory areas. These findings align with the established network involved in encoding episodic memory, where sensory inputs register across primary sensory areas before being processed through association areas. Extensive significant associations of all MAP‐MRI parameters with the Trail Making test scores were found in attention, self‐processing, and visual network regions, in agreement with the observed decline with age based on neuropsychological studies (Tombaugh, 2004). Using the RAVLT, which is widely applied for cognitive assessment of memory consolidation in normal aging, pre‐dementia, and dementia conditions, negative associations of PA with retroactive interference were found in auditory processing and integration networks. These regions, along with memory‐related regions, exhibited negative correlations of NG and PA with proactive interference.

Despite the study's strengths, it has limitations. The large number of subjects is beneficial, but they were mainly white, highly educated individuals from the HCP‐A database, limiting generalization due to potential biases related to race, ethnicity, culture, and education. Being cross‐sectional, the study lacks a longitudinal design, but future analysis of the forthcoming HCP‐A follow‐up data could enhance its scope. A longitudinal design would allow to directly determine the order in which micro‐ and macrostructural changes take place across the lifespan, and whether earlier changes can predict the development of later changes and cognitive decline. The age cohort, starting from 36 years, restricts exploration of changes across the age spectrum, potentially emphasizing late maturation and degeneration phases. In addition, the age‐related changes in microstructure and cognition could be partially confounded by the presence of any preclinical Alzheimer's disease pathology, which was not extensively evaluated in the HCP‐A cohort. While the HCP‐A dMRI dataset provides good orientational coverage and AP‐PA encoding, the maximal b‐value is 3000 s/mm^2^. The relatively low diffusion sensitization in our study limits our ability to capture subtle features of the underlying diffusion propagators. Consequently, we measured the propagators using 22 coefficients associated with the most significant basis functions in a MAP‐MRI series expansion truncated at order 4 (Özarslan et al., 2013). The inclusion of higher b‐values, up to 6000 s/mm^2^ (Huang et al., 2021), would have allowed us to consider terms up to order 6, as found to be optimal in clinical applications (Avram et al., 2016). Subsequent studies ought to explore and delineate microscopic alterations in the shape, dimensions, and geometry of cerebral tissue throughout the lifespan. While the conventional linear diffusion encoding scheme that is used here does not allow the decoupling of size and microscopic orientation, utilizing multiple diffusion encoding schemes such as planar (Mitra, 1995) or spherical (Topgaard, 2017) is recommended to study these effects. Additionally, the simultaneous application of relaxation and diffusion encoding should be considered in future investigations (Benjamini et al., 2023; Martin et al., 2021).

In conclusion, this comprehensive analysis utilizes a large cross‐sectional dataset and applies the advanced MAP‐MRI model to delineate GM microstructural relationships with age and neurobehavioral performance across healthy aging. Notably, while we did not identify macrostructural volumetric associations with neurobehavioral scores, we did, for the first time, establish robust correlations between GM microstructure and age‐related neurobehavioral performance. Based on our findings, we hypothesize that increased microstructural heterogeneity and decreased neurite orientation dispersion precede macrostructural changes, and that they play an important role in subsequent cognitive decline. This study provides valuable insights that could assist in the early differentiation of cognitively healthy aging from pathological conditions, including mild cognitive impairment, Alzheimer's, and other dementia‐related disorders.

## 4 METHODS

### 4.1 Study design and participants

Data were obtained from Human Connectome Project‐Aging (HCP‐A) Lifespan 2.0 Release. It included 725 healthy adults aged 36 to 90+ years old acquired across four acquisition sites using matched MRI scanning protocols (Harms et al., 2018). All participants were screened for causes of cognitive decline and exhibited typical health for their age without stroke, clinical dementia. The study was approved and monitored by the Institutional Review Board. Written informed consent was obtained from all participants in the study. After written consent, the Montreal Cognitive Assessment (MoCA) was administered, and participants meeting the determined normal threshold for their age bracket were considered eligible for the study. No participants were excluded based on medication use, although self‐reported medication use was recorded during the study visit to investigate or avoid specific medication confounds. Essential health assessments known to show associations to brain circuitry during typical aging, as well as in dementia and other diseases, was performed and has been previously described (Bookheimer et al., 2019). This process excluded participants who have been diagnosed and treated for major psychiatric disorders (e.g., schizophrenia and bipolar disorder) or neurological disorders (e.g., stroke, brain tumors and Parkinson's disease).

Of 725 healthy subjects' data available, we included 707 subjects for MAP‐MRI, DTI, and volumetric analysis based on quality control of the raw data. The study population demographics is provided in Table 1. During HCP‐A 2.0 release, data from participants over the age of 90 years were lumped together due to data policy, and therefore were excluded from our study. Years of education were also recorded and used in the final analysis.

### 4.2 Imaging data acquisition

To meet the HCP‐A recruitment and diversity goals, data were acquired at four different institutions. In all four sites, data were acquired using a 3T scanner (MAGNETOM Prisma, Siemens Healthcare AG, Erlangen, Germany) with a 32 channel head coil.

Structural MRI images were acquired using T1‐ and T2‐weighted (T1W and T2W) contrasts. T1‐weighted imaging was performed using a multi‐echo MPRAGE sequence with 0.8 mm isotropic voxel size, TR = 2500 ms, TI = 1000 ms, TE = 1.8/3.6/5.4/7.2 ms, and flip angle of 8°. T2‐weighted imaging was performed using a T2w‐SPACE protocol with 0.8 mm isotropic voxel size, TR = 3200 ms and TE = 56.4 ms. Both sequences used embedded volumetric navigators for prospective motion correction and selective reacquisition of the lines in k‐space corrupted by motion. The mean image of just the first two echoes from the MPRAGE acquisition was used as the input to subsequent processing.

Diffusion‐weighted images (DWIs) were acquired using a pulsed gradient spin‐echo sequence with 1.5 mm isotropic voxel size and TR/TE = 3230/89.5 ms. Diffusion encoding was acquired with two shells of 1500 and 3000 s/mm^2^ (98–99 directions per shell), and with 28 b‐value = 0 s/mm^2^ images interleaved. All datasets were acquired with two phase encoding directions: anterior to posterior (AP), and reversed phase encoding direction (PA). More detailed image acquisition protocols of HCP‐A can be found in Harms et al (Harms et al., 2018).

### 4.3 Behavior and cognitive testing

The HCP‐A conducted detailed behavioral and cognitive assessments using NIH toolbox. The list of tests and respective scores stratified by participants demographics is provided in Table 1. Briefly, the tests included in the current study are as follows: (1) Flanker Inhibitory Control and Attention Test, which is an assessment of inhibitory control and attention. The participant is asked to focus on a particular stimulus while inhibiting attention to the stimuli flanking it. (2) Picture Sequence Memory (PSM) test, which assess episodic memory. Participants are shown several pictures, and then asked to reproduce the sequence of pictures as it was presented to them. (3) The Trail Making Test is a neuropsychological test of visual attention and task switching, in which Trail Making A (connecting numbers sequentially) measures cognitive processing speed, whereas Trail Making B (connecting numbers and letters sequentially) measures executive functioning. (4) For a more comprehensive assessment of episodic memory, a widely used neuropsychological measure, the Rey Auditory Verbal Learning Test (RAVLT) was used. The RAVLT consists of a semantically unrelated word list (List A) and a similar interference list (List B). Participants are given five trials to immediately recall as many words as possible from list A of 15 unrelated words. On the next trial, subjects are asked to immediately recall words of interference list (List B). For the final trial (Trial 6 for list A), the participant is asked to recall as many words as they can from List A. To interpret these scores, we computed RAVLT 1, which is the relative difference between trial 6‐list A and trial 5‐list A, and RAVLT2, which is the relative difference between list B and trial 1‐list A. Low RAVLT 1 score indicative of either high degrees of forgetting during the short delay or retroactive interference, and low RAVLT 2 score indicative of a high degree of proactive interference.

### 4.4 Data processing

#### 4.4.1 Preprocessing

Each participant DWIs were manually quality checked before and during each processing step. The preprocessing modules used in this work are part of the TORTOISE dMRI processing package (Irfanoglu et al., 2023). Briefly, the dMRI data initially underwent denoising with the MPPCA technique (Veraart et al., 2016), which was followed by Gibbs ringing correction for partial k‐space acquisitions. Motion and eddy currents distortions were subsequently corrected with TORTOISE's DIFFPREP module with a physically‐based parsimonious quadratic transformation model and a normalized mutual information metric. The final preprocessed data was output with a single interpolation in the space of an anatomical image at native in‐plane voxel size.

### 4.5 DTI parameters estimation

DTI was computed with nonlinear regression. The scalar maps of choice for the current study were fractional anisotropy (FA), axial diffusivity (AD), radial diffusivity (RD), and diffusion trace (TR) from the tensor model.

### 4.6 MAP‐MRI parameters estimation

Using these preprocessed DWIs, we estimated the voxel‐wise diffusion propagators using a MAP‐MRI series expansion truncated at order 4 (Özarslan et al., 2013). MAP‐MRI extends the DTI model, harnessing the capabilities of modern dMRI sequences to offer potentially more sensitive metrics for detecting early pathological events in the disease process (Avram et al., 2016) and to quantify microscopic flow (Benjamini et al., 2019). The commonly derived metrics are the propagator anisotropy (PA), which is a generalized version of DTI's fractional anisotropy (FA); the non‐Gaussianity (NG), which quantifies the dissimilarity between the propagator and its Gaussian part; and the zero displacement probabilities, including the return to the origin probability (RTOP), the return to the axis probability (RTAP), and the return to the plane probability (RTPP), which comprehensively quantify various features of the three‐dimensional diffusion process. We therefore computed images of the MAP parameters: RTOP, RTAP, RTPP, NG, and PA. Note that throughout the paper we report the RTAP^1/2^ and RTOP^1/3^ values to allow consistency in units of the zero‐displacement probability metrics (i.e., 1/mm).

### 4.7 Whole brain segmentation and volume estimation

The spatially localized atlas network tiles (SLANT) method was used to perform whole brain segmentation (Huo et al., 2019). Briefly, SLANT employs multiple independent 3D convolutional networks for segmenting the brain. Each of the networks is only responsible for a particular spatial region, thus the task of each network is simplified to focus on patches from a similar portion of the brain. Within this end‐to‐end pipeline, affine registration, N4 bias field correction, and intensity normalization are employed to roughly normalize each brain to the same space before segmentation. After each network performs its duty, the segmentation labels are fused together to form the labels for 132 anatomical regions based on the BrainCOLOR protocol (https://mindboggle.info/braincolor/). For this study, we first merged the right and left side brain labels. Out of these labels, we then excluded WM and ventricles regions, and regions that might give nonspecific information due to large size (e.g., cerebellum and brainstem). Finally, a total of 56 ROIs were included in the study, as shown in Figure 1. These labels were used, after adjusting for intracranial volume (ICV) to remove the confounding effect of head size, to obtain regional brain volumes. For statistical analysis, SLANT labels were first transformed from T1W space to T2W/DWI space using ANTs rigid registration. Additionally, all ROIs were eroded using a 2 × 2 × 2 voxels cubic structuring element to reduce partial volume effects and imperfect image registration and to mitigate structural atrophy seen especially at older ages. The mean NG, PA, RTAP, RTOP, and RTPP values were calculated for each ROI and participant.

### 4.8 Statistical analysis

To investigate micro‐ and macrostructural changes in the brain due to aging, multiple linear regression was applied on the MAP metrics (i.e., NG, PA, RTAP, RTOP, and RTPP), DTI metrics (i.e., FA, AD, RD, TR), and ICV. These 10 MRI features along with the behavioral and cognitive test scores were z‐normalized for further analysis.

We first assessed effect of age on MRI parameters using an age quadratic model, with each mean MRI metric within each ROI as the dependent variable. The model is given by:

$$\begin{array}{c} P_{i}=\beta_{0}+\beta_{\mathrm{sex}}\times \mathrm{sex}+\beta_{\mathrm{age}}\times \mathrm{age}+\beta_{\mathrm{age}}^{2}\times age^{2}\text{+}\beta_{\mathrm{YOE}}\times \mathrm{YOE}\\ +\beta_{\mathrm{vol}}\times \text{Volume}+\beta_{\text{site}}\times \text{site}+\beta_{\text{inter}}\times sex\times \mathrm{age}+\beta_{\text{inter2}}\times sex\times age^{2}, \end{array}$$

where 𝑃_𝑖_ is the mean ROI value of the parameter of interest (e.g., NG, PA, volume, etc.) of the *i*th ROI. Sex, years of education (YOE), total brain volume (Volume), and sex–age interactions were accounted for. In addition, we included a categorical *site* variable as a covariate to account for the four HCP‐A data acquisition locations. Results are presented as the beta coefficients of estimates 𝛽_age_ and 𝛽_age_^2^, which due to standardization represents the standard deviation change in MR variable per year. To estimate the peak age for each MR metric and within each region we equated the first derivative of the quadratic function to zero, such that the peak age was given by −𝛽_age_/2𝛽_age_^2^. To avoid extrapolation beyond the extent of our data, we left censored the peak age at 36. The standard error for the estimated peak ages was estimated using the delta method, which resulted in median values across all ROIs of 11.0, 9.4, 3.2, 3.4, 3.0, and 6.0 years for NG, PA, RTAP, RTOP, RTPP, and Volume, respectively.

In our second model, cognitive test scores were used as outcomes,

$$C=\beta_{0}+\beta_{\mathrm{sex}}\times \mathrm{sex}+\beta_{\mathrm{age}}\times \mathrm{age}+\beta_{\mathrm{YOE}}\times \mathrm{YOE}+\beta_{\mathrm{vol}}\times \text{Volume}+\beta_{\mathrm{MR}}\times P_{i},$$

where *C* are the neurobehavioral test scores (i.e., Flanker, PSM, etc.). In this model, cognitive test scores predictors and the MR response variable were standardized because they all have different scales. In this case, the coefficients 𝛽_MR_ could be interpreted as the change in the response variable (in standard deviations) for a 1 standard deviation change in the predictor.

We mean centered age variable and used effect coding for sex (0.5 for males and −0.5 for females). These centering methods allow regression coefficient to have meaningful interpretation in the presence of interaction or quadratic terms. False discovery rate (FDR) correction was done to correct for multiple comparisons (Storey, 2002) and the threshold for statistical significance was *p*_FDR_ <0.05.

In addition, to show the effect size of different dependent variables, we report partial Pearson correlation coefficients adjusted for the covariates.

Matlab was used for all computations.

## AUTHOR CONTRIBUTIONS

D.B. conceived the study; D.B. and K.S. obtained the data; K.S., S.B., and K.G.S. analyzed the data; Y.A. designed the statistical models; L.F. contributed to the interpretation of the results and worked on the manuscript; K.S. and D.B. drafted the manuscript. All authors interpreted the findings, commented on the manuscript, and approved the submitted version.

## CONFLICT OF INTEREST STATEMENT

The authors declare no competing financial interests.

## Supporting information


Figures S1–S14.

## Data Availability

The data that support the findings of this study are openly available in the Human Connectome Project at https://www.humanconnectome.org/.
