# Supplementary material for: Neuronal microstructural changes in the human brain are associated with neurocognitive aging
Source: Aging Cell. 2024 Apr 24;23(7):e14166. doi: 10.1111/acel.14166 (PMC11258428; doi:10.1111/acel.14166)
Supplement: Supplementary file 1 — Figures S1–S14. [file ACEL-23-e14166-s001.pdf]

Supplementary Information: Neuronal microstructural changes in the human brain are associated with neurocognitive aging

Kavita Singh[1*], Stephanie Barsoum[1], Kurt G Schilling[2], Yang An[3], Luigi Ferrucci[4], Dan Benjamini[1*]

Affiliations:

[1]Multiscale Imaging and Integrative Biophysics Unit, National Institute on Aging, NIH, Baltimore, MD, USA

[2]Department of Radiology and Radiological Sciences, Vanderbilt University Medical Center, Nashville, TN, USA

[3]Brain Aging and Behavior Section, National Institute on Aging, NIH, Baltimore, MD, USA

[4]Translational Gerontology Branch, National Institute on Aging, NIH, Baltimore, MD, USA

*Correspondence to:
Dan Benjamini, PhD
The National Institute on Aging
251 Bayview Blvd. Baltimore, MD 21224, USA
Email: dan.benjamini@nih.gov

Correspondence may also be sent to:
Kavita Singh, PhD
The National Institute on Aging
251 Bayview Blvd. Baltimore, MD 21224, USA
Email: kavita.singh3@nih.gov

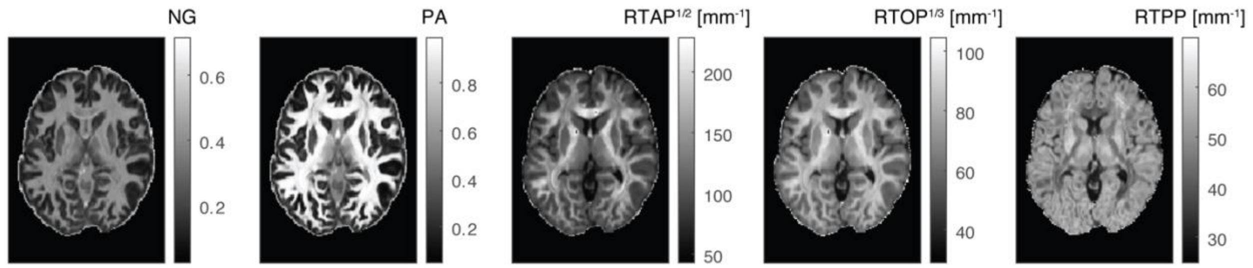

Supplementary Figure 1: MAP metrics maps from a representative subject.

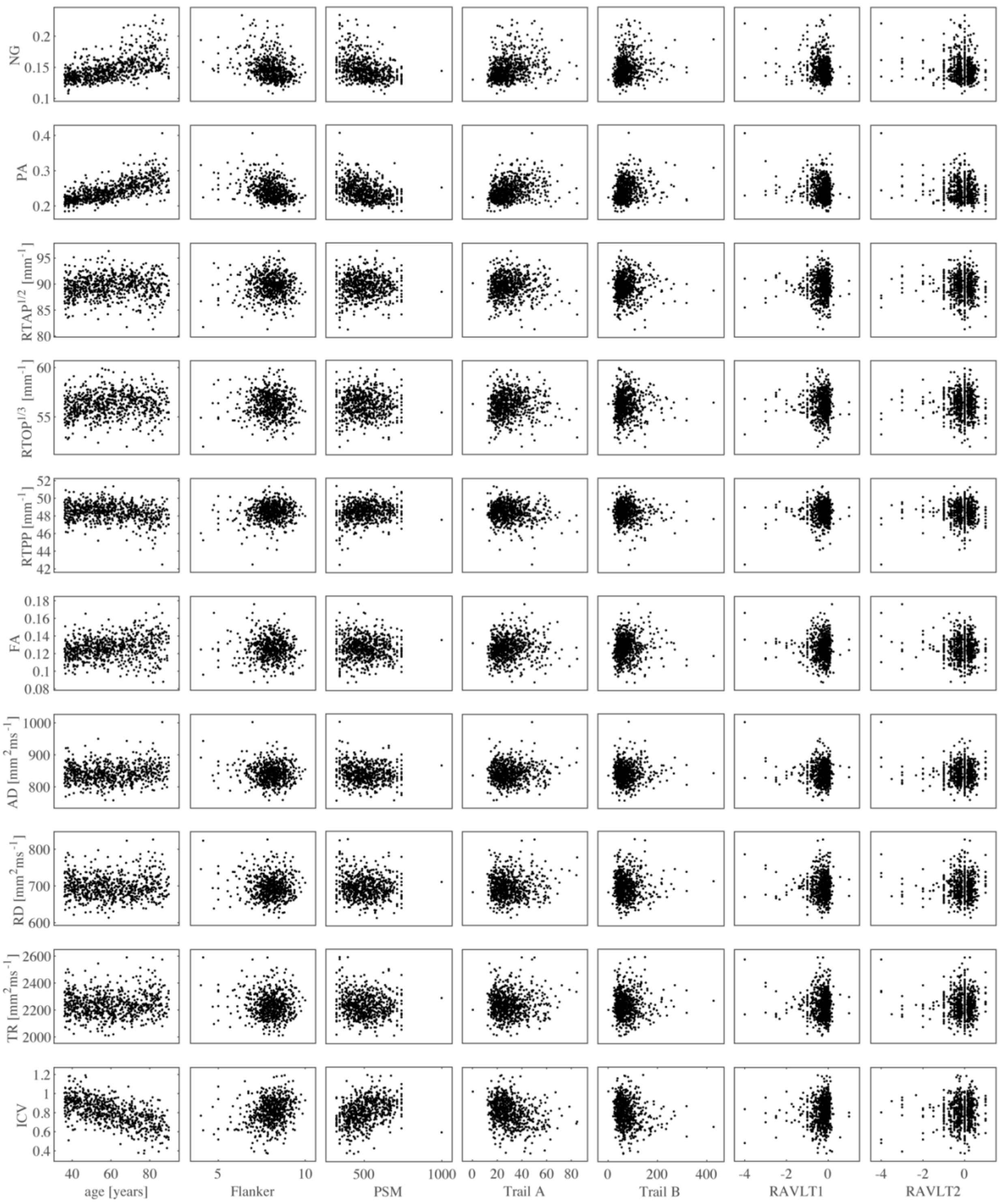

Supplementary Figure 2: Scatter plots of all MR parameters with respect to age, Flanker, PSM, Trail Making A, Trail Making B, RAVLT1 and RAVLT2 test scores in the medial superior frontal gyrus.

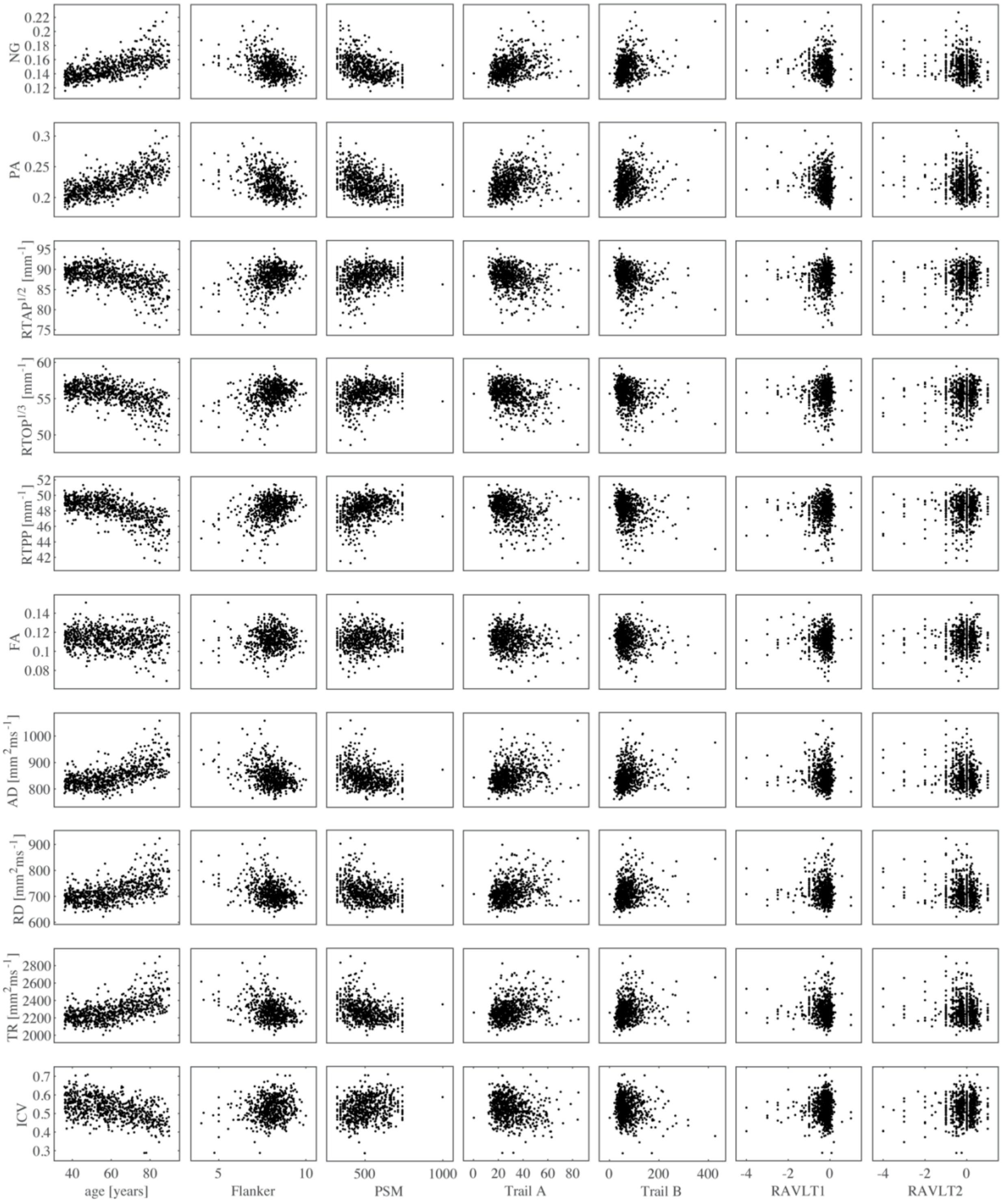

Supplementary Figure 3: Scatter plots of all MR parameters with respect to age, Flanker, PSM, Trail Making A, Trail Making B, RAVLT1 and RAVLT2 test scores in the central operculum.

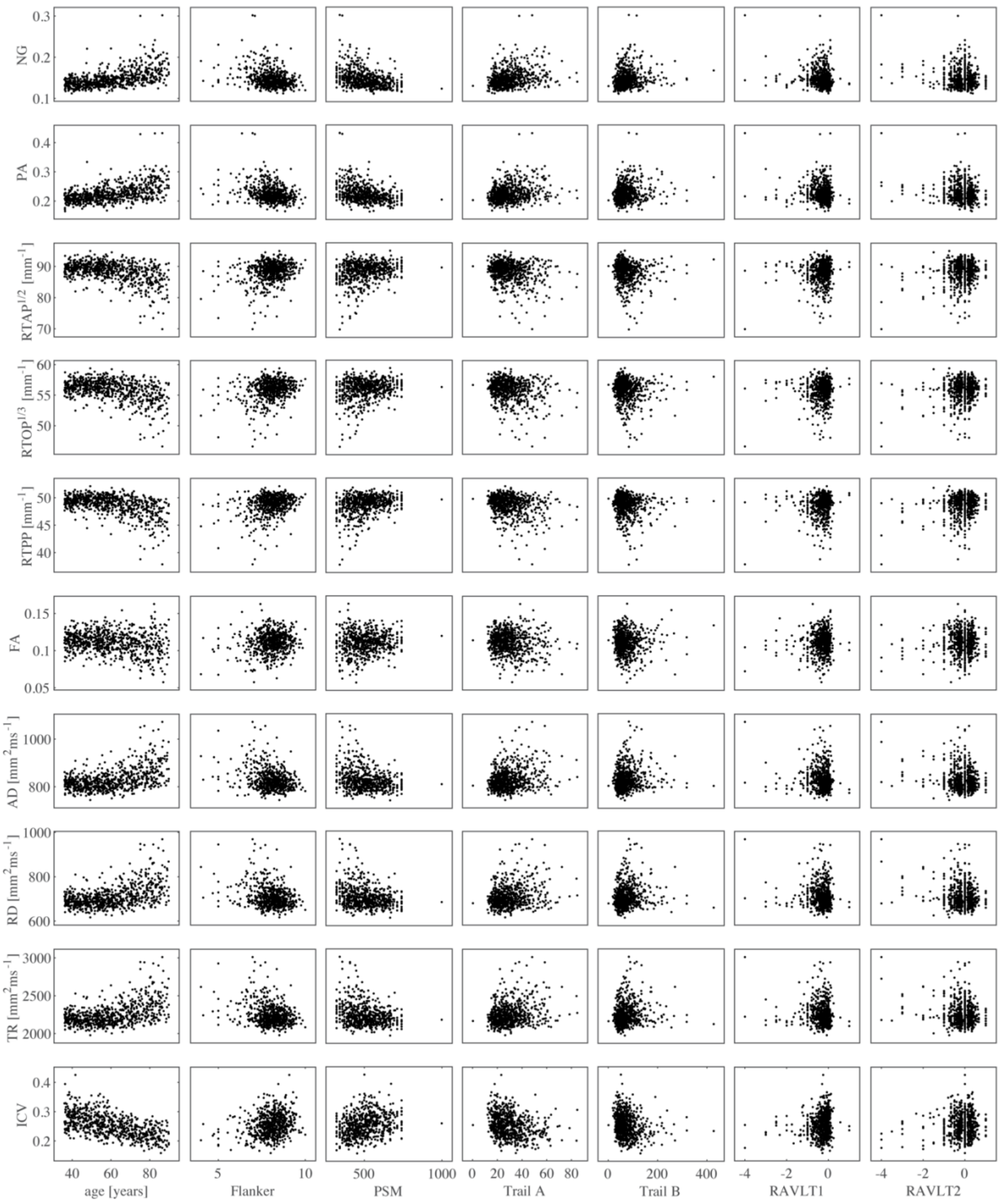

Supplementary Figure 4: Scatter plots of all MR parameters with respect to age, Flanker, PSM, Trail Making A, Trail Making B, RAVLT1 and RAVLT2 test scores in the planum polare.

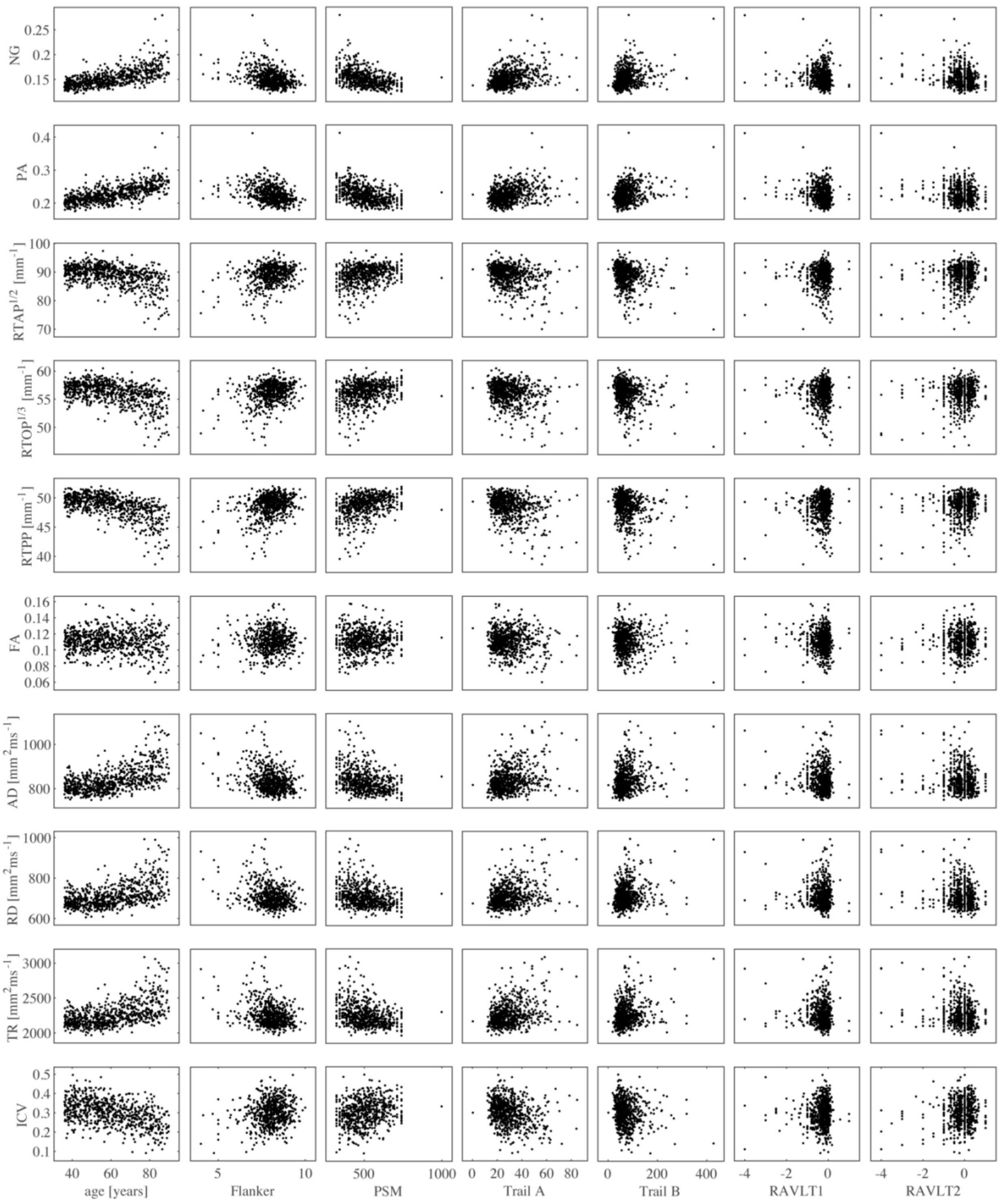

Supplementary Figure 5: Scatter plots of all MR parameters with respect to age, Flanker, PSM, Trail Making A, Trail Making B, RAVLT1 and RAVLT2 test scores in the parietal operculum.

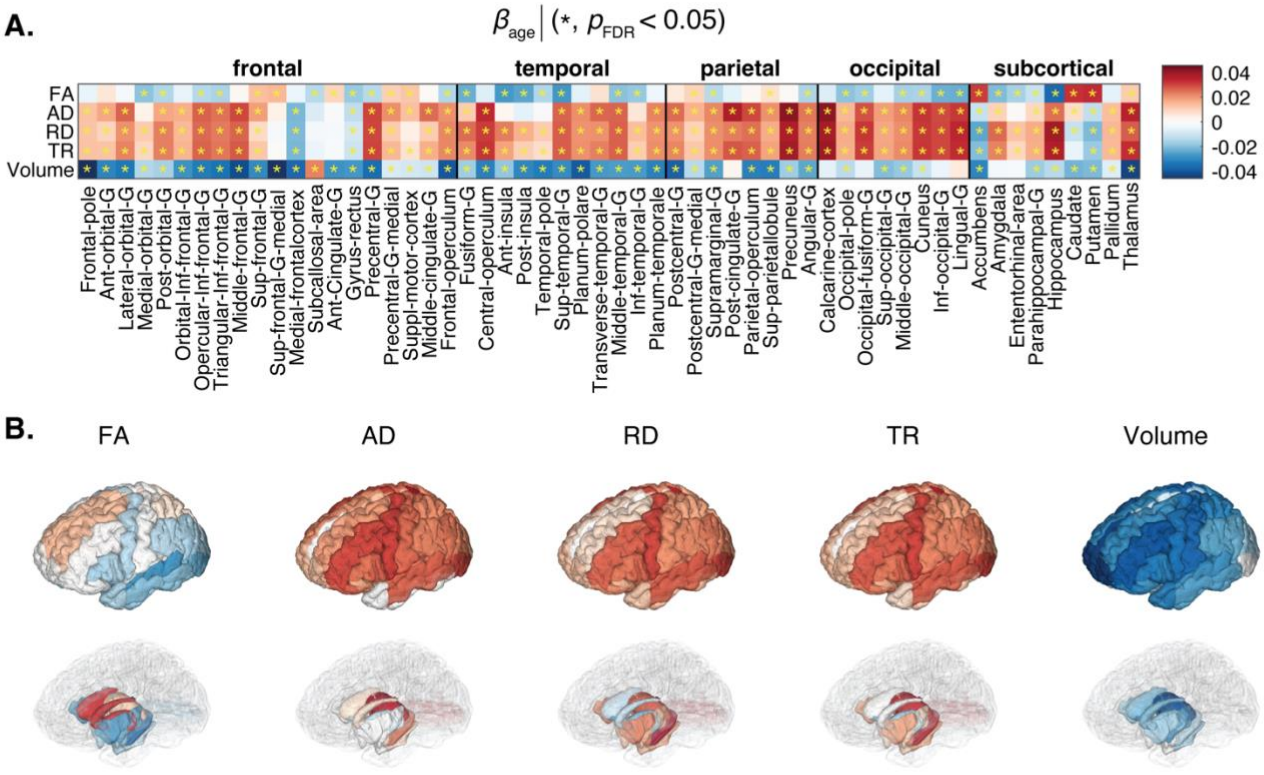

Supplementary Figure 6: Linear associations of DTI and volumetric metrics and age. (A) The β_{age} coefficients are shown as a matrix for DTI and volumetric z-normalized features across all 56 ROIs. Blocks marked with an asterisk (*) represent associations meeting the p_{FDR}<0.05 threshold. (B) 3D visualization of significant results in cortical (top) and subcortical (bottom) view.

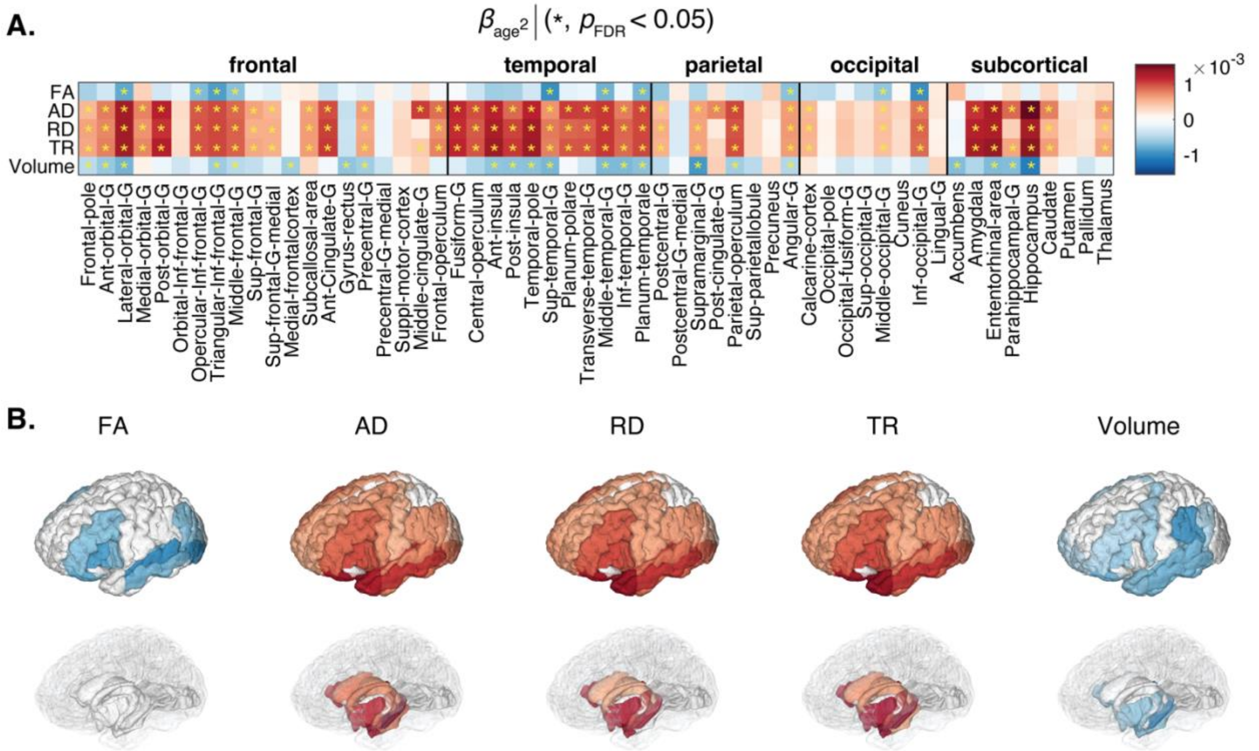

Supplementary Figure 7: Quadratic associations of DTI and volumetric metrics and age. (A) The β_{age^2} coefficients are shown as a matrix for DTI and volumetric z-normalized features across all 56 ROIs. Blocks marked with an asterisk (*) represent associations meeting the p_{FDR}<0.05 threshold. (B) 3D visualization of significant results in cortical (top) and subcortical (bottom) view.

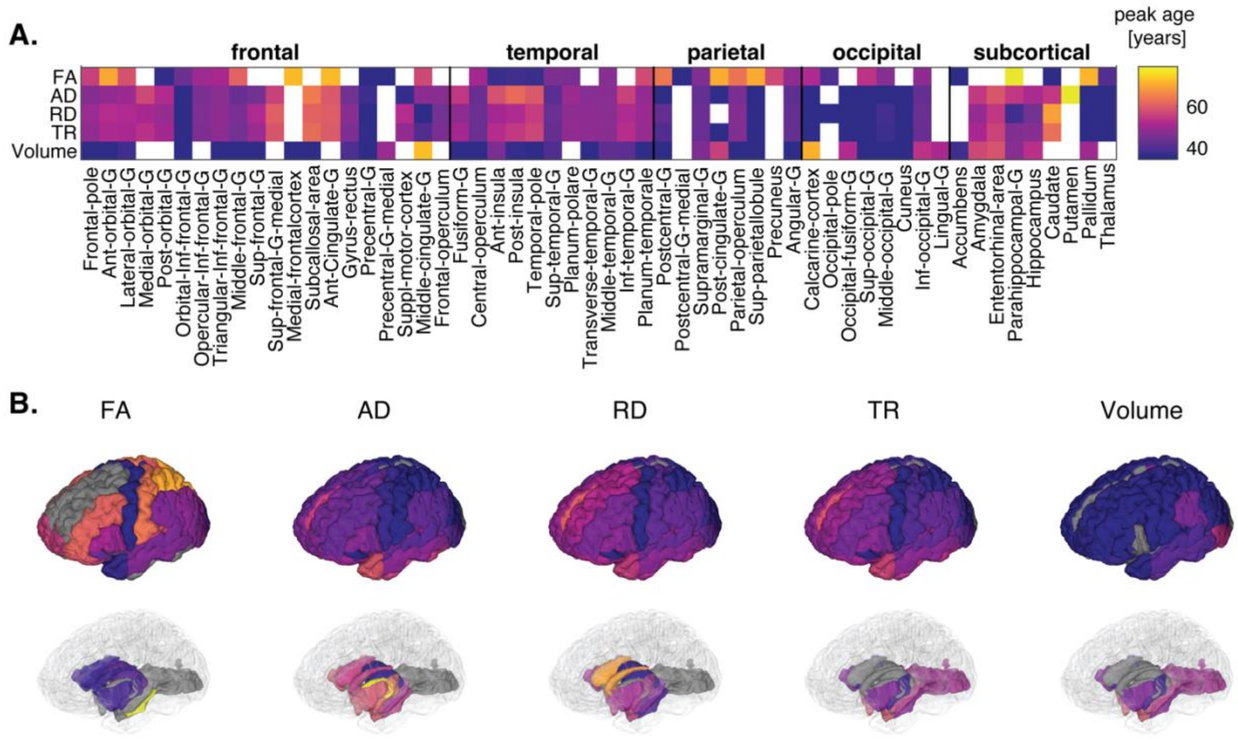

Supplementary Figure 8: Peak ages for DTI and volumetric within each ROI. (A) Matrix representation of peak age across all 56 ROIs. (B) 3D visualization of significant peak ages in cortical (top) and subcortical (bottom) view.

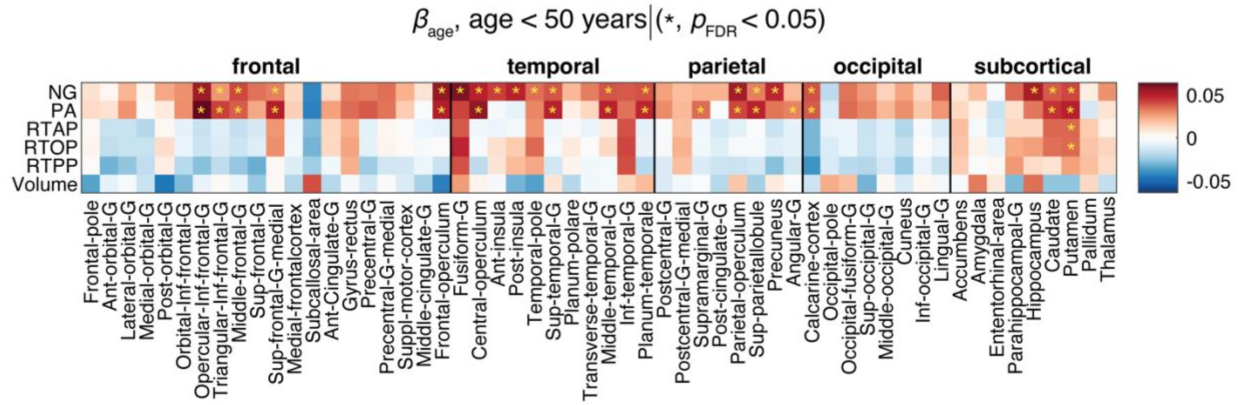

Supplementary Figure 9: Linear associations of MAP-MRI and volumetric metrics and age in a subset of subjects under the age of 50 (N=227). The β_{age} coefficients are shown as a matrix for MAP-MRI and volumetric z-normalized features across all 56 ROIs. Blocks marked with an asterisk (*) represent associations meeting the p_{FDR}<0.05 threshold.

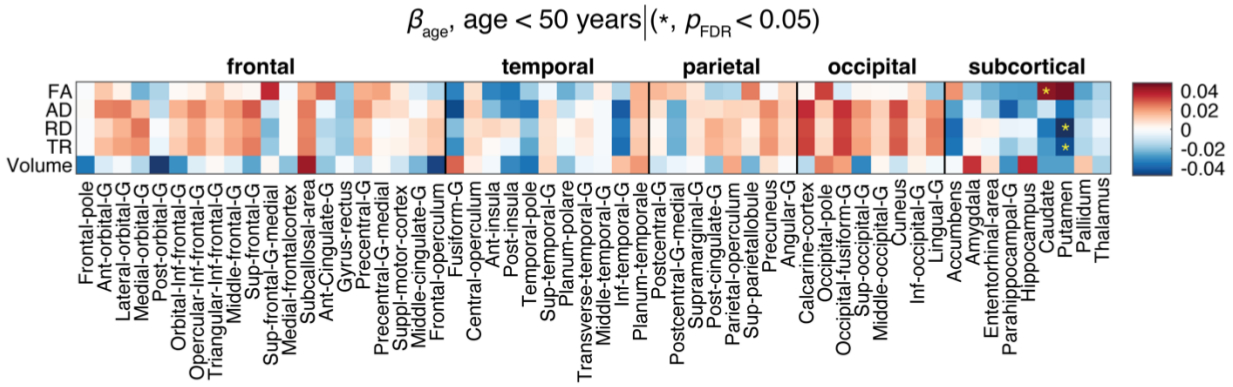

Supplementary Figure 10: Linear associations of DTI and volumetric metrics and age in a subset of subjects under the age of 50 (N=227). The β_{age} coefficients are shown as a matrix for DTI and volumetric z-normalized features across all 56 ROIs. Blocks marked with an asterisk (*) represent associations meeting the p_{FDR}<0.05 threshold.

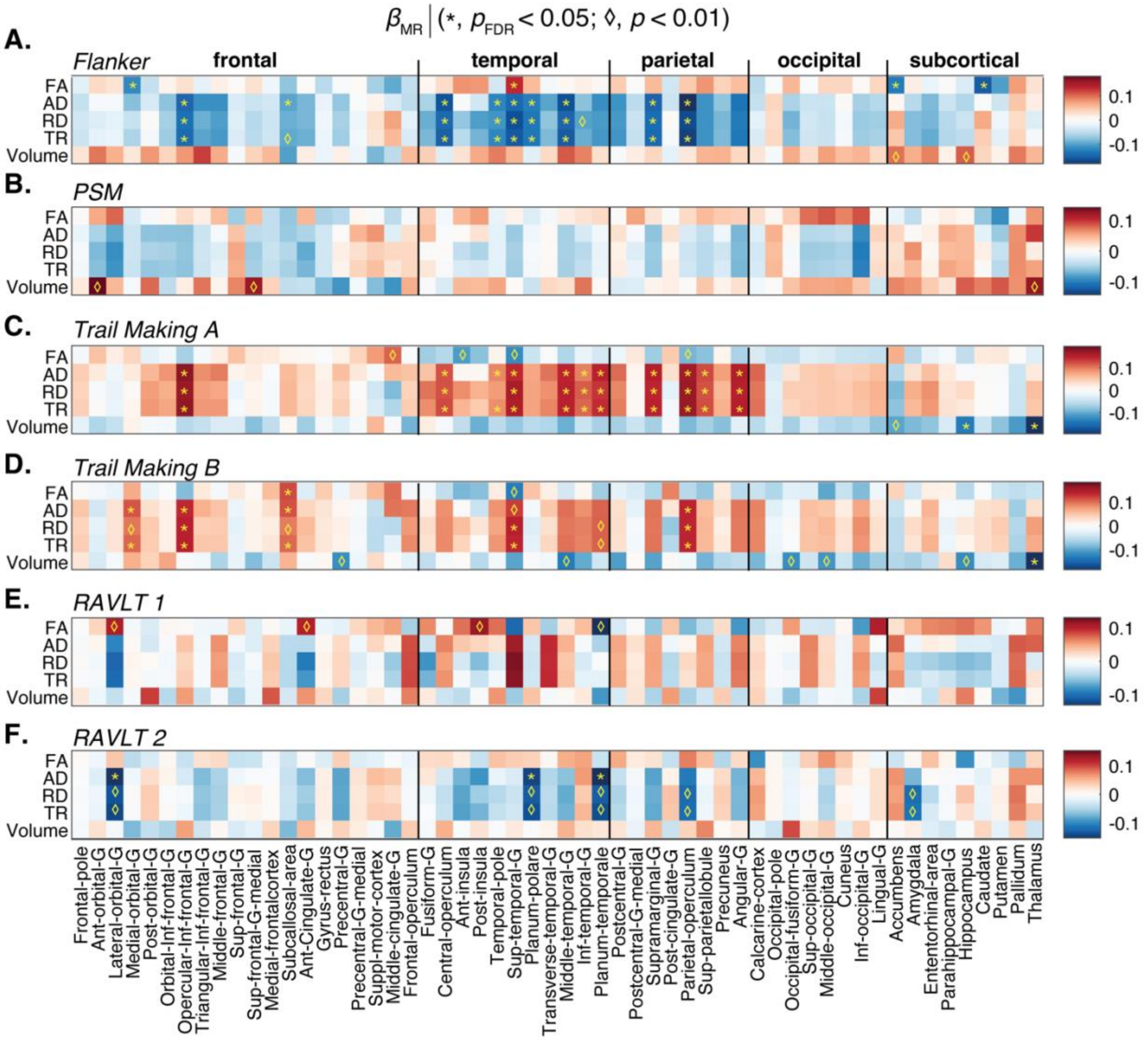

Supplementary Figure 11: Significant results of second statistical model examining the relationship between volumetric and DTI metrics and six cognitive test scores by modeling them as outcomes, adjusted for age, sex and years of education. Significant associations are expressed using the regression parameters β_{MR}. Blocks marked with an asterisk (*) represent associations meeting the p_{FDR}<0.05 threshold. Blocks marked with a diamond (◊) represent associations meeting the p<0.01 threshold without FDR correction. For all tests, the lower the score the worse the performance, except for the Trail Making task, in which the opposite is true. PSM; Picture Sequence Memory test; RAVLT: Rey auditory verbal learning test.

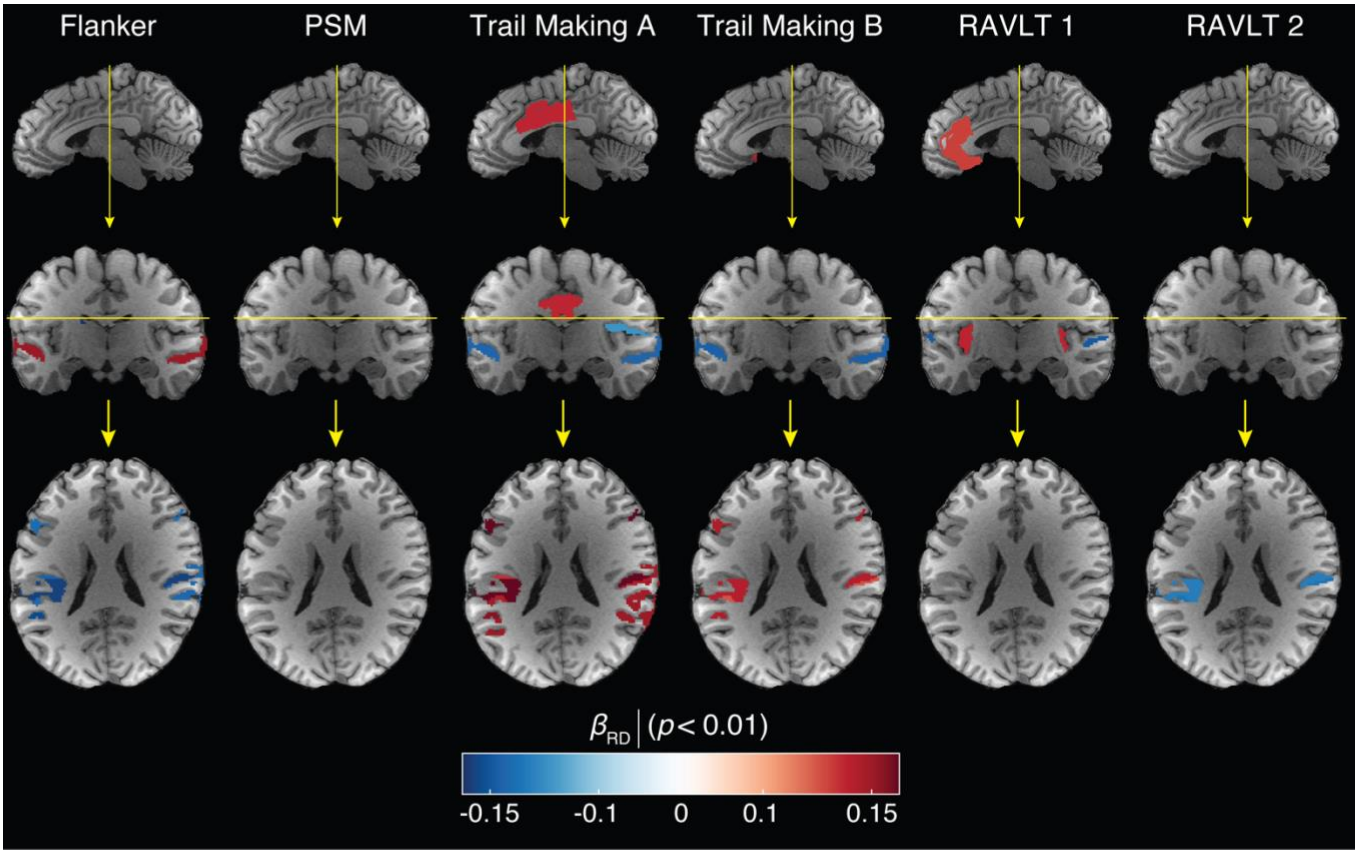

Supplementary Figure 12: Axial, coronal, and sagittal views of significant results of relationship between the radial diffusivity (RD) and 6 cognitive test scores. Age-related significant associations are expressed using the regression parameters β_{NG} with $p < 0.01$. For all tests, the lower the score the worse the performance, except for the Trail Making task, in which the opposite is true. PSM: Picture Sequence Memory test; RAVLT: Rey auditory verbal learning test. Yellow lines indicate sectional planes.

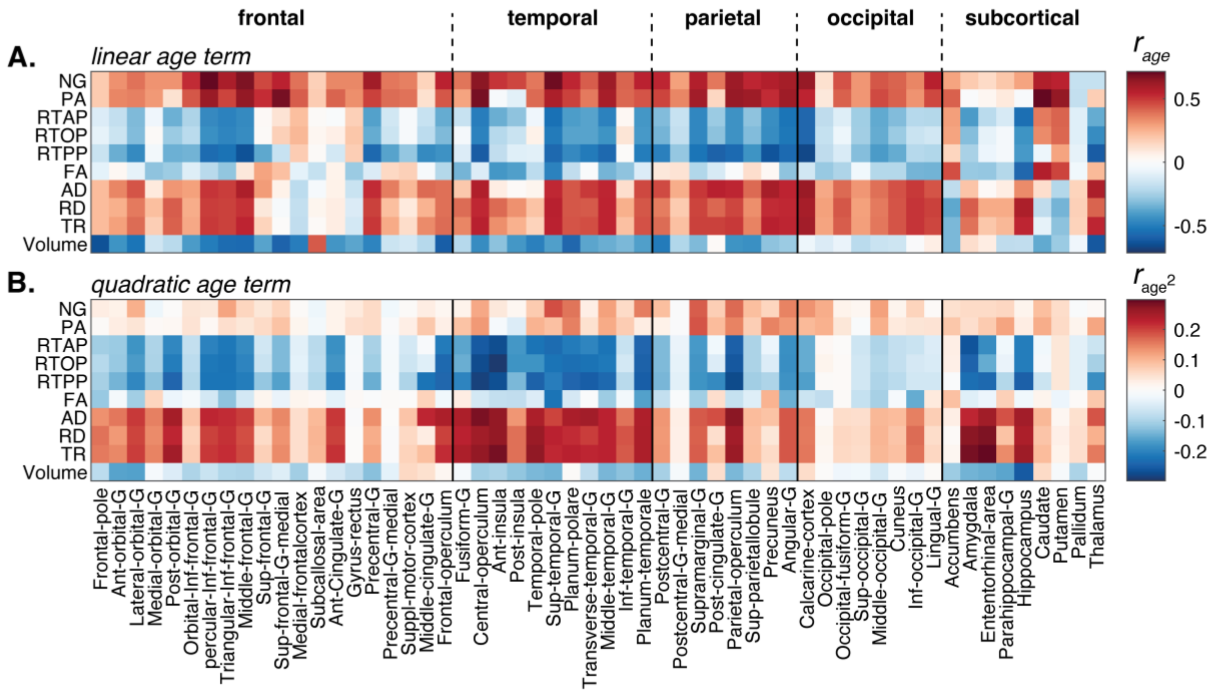

Supplementary Figure 13: Partial Pearson correlation coefficients examining the effect size between MRI parameters and the (A) linear and (B) quadratic age terms.

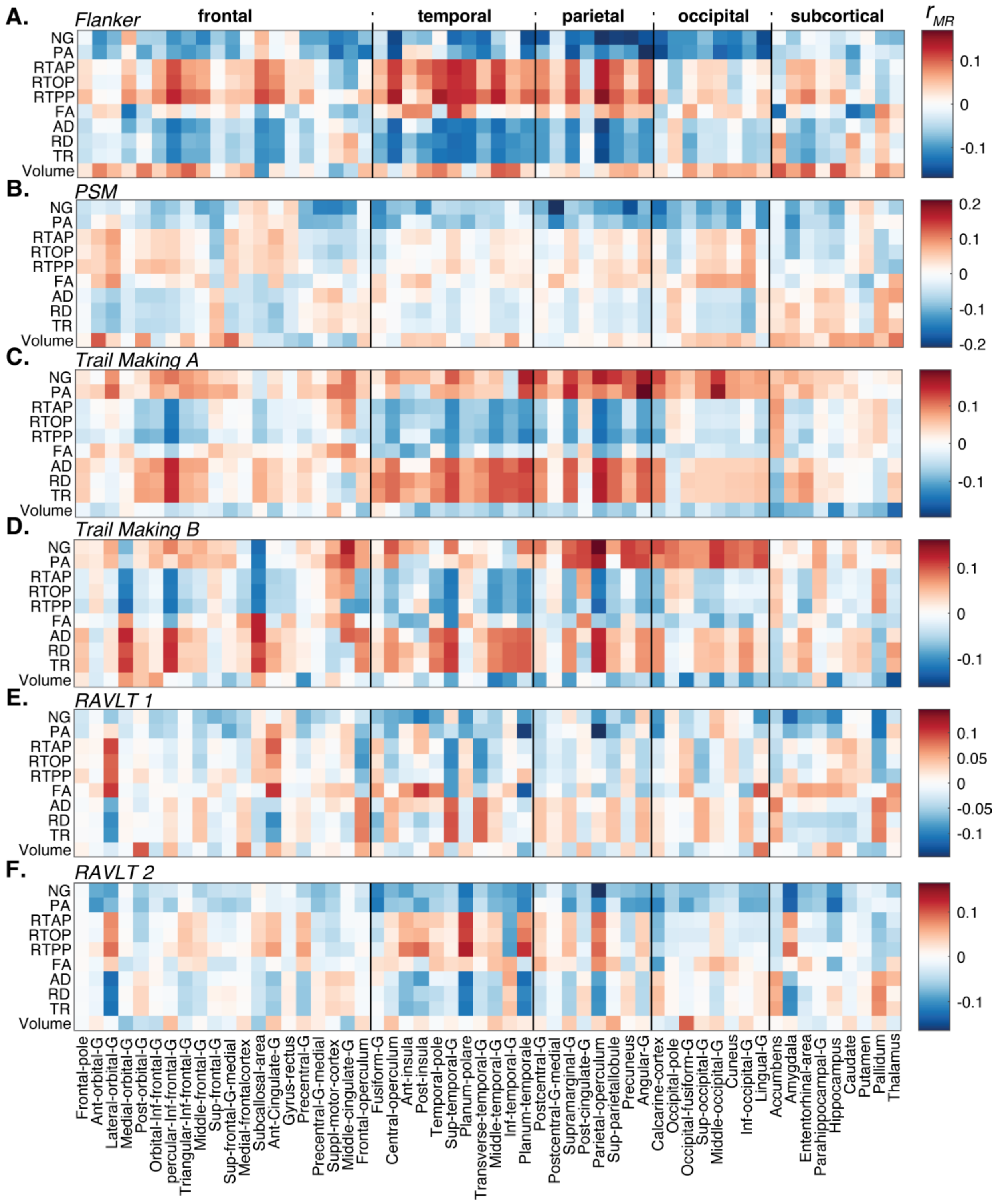

Supplementary Figure 14: Partial Pearson correlation coefficients examining the effect size between MRI parameters and six cognitive test scores, adjusted for age, sex and years of education. PSM; Picture Sequence Memory test; RAVLT: Rey auditory verbal learning test.
